# Jejunal mucosal immune response in goats ten months after *Mycobacterium avium* subsp. *paratuberculosis* challenge is primarily determined by lesion severity rather than vaccination route

**DOI:** 10.1080/01652176.2026.2707278

**Published:** 2026-07-23

**Authors:** Miguel Criado, Marta Silva, Pedro Mendívil, David Zapico, Victoria Pérez-Rojo, Julio Benavides, José Espinosa, Natalia Elguezabal, Daniel Gutiérrez-Expósito

**Affiliations:** a Departamento de Sanidad Animal, Facultad de Veterinaria, Universidad de León, León, Spain; b Departmento de Sanidad Animal, Instituto de Ganadería de Montaña, CSIC-ULE, León, Spain; c Departamento de Sanidad Animal, NEIKER-BRTA, Instituto Vasco de Investigación y Desarrollo Agrario, Derio, Spain

**Keywords:** *Paratuberculosis*, mucosal immunity, vaccination route, oral vaccination, intradermal vaccination, *Mycobacterium avium* subsp. *paratuberculosis*, cytokines, Th1/Th2 polarization, Secretory IgA

## Abstract

Current vaccines against *paratuberculosis* offer incomplete and variable protection that raises questions on the ruminant immune responses to *Mycobacterium avium* subspecies *paratuberculosis* (*Map*) infection, particularly at the mucosal level. Thus, this study examined the immune responses established in the jejunum of goats vaccinated with oral, intradermal, or subcutaneous inactivated vaccines 11 months post-vaccination and 10 months after experimental *Map* challenge. In general terms, the mucosal response of non-challenged animals was not affected by vaccination. However, the quantification of immune cell populations, cytokine expression, recall responses of tissue-isolated mononuclear leukocytes, and secretory IgA levels in vaccinated and challenged animals with focal lesions suggested a local Th1 polarization. In contrast, those with diffuse lesions exhibited features compatible with the early development of a more Th2-biased response. The vaccination route played a lesser role in the immune response than lesion severity. Orally vaccinated and challenged goats, showed reduced local IL-1β and IL-17A expression, which may explain the milder lesions observed in these animals, despite their high bacterial burden, as these cytokines participate in macrophage recruitment, granuloma formation, and bacterial clearance. Nevertheless, owing to the high variability among challenged animals, larger sample sizes would allow robust conclusions to be drawn in further studies.

## 1. Introduction

*Paratuberculosis* (PTB) caused by the infection of *Mycobacterium avium* subspecies *paratuberculosis* (*Map*) is a slow disease characterized by a progressive granulomatous enteritis, which affects ruminants worldwide, increasing mortality and reducing production in affected herds (Lombard 2011). Vaccination is considered the most effective strategy for controlling clinical disease, but it has several drawbacks, including interference with bovine tuberculosis (bTB) diagnosis, the development of a large granuloma in the injection site and failure to provide complete protection (Bastida and Juste 2011; de Silva et al. 2015; Criado et al. 2024). To overcome these drawbacks, multiple approaches have been explored. Alternative strategies to the whole-cell inactivated vaccines currently used for PTB control have been developed and tested, including attenuated *Map* strains (Bannantine et al. 2014), subunit (Koets et al. 2006), and DNA-based vaccines (Roupie et al. 2012). Additionally, an increasing number of alternatives to mineral oil adjuvants, which are responsible for granuloma formation at the injection site (Tizard 2021), have been evaluated (Begg et al. 2019; Ladero-Auñon et al. 2021). While alternative immunization routes and delivery methods have been widely investigated for other human (Criscuolo et al. 2019) and veterinary (Wilson et al. 2020) vaccines—particularly for bTB (Buddle et al. 2018; Juste et al. 2025)—, they have received comparatively little attention in PTB vaccination (Arrazuria et al. 2020; Eshraghisamani et al. 2023).

Currently available PTB vaccines (Gudair® and Silirum®) are whole-cell, heat-killed preparations formulated in an oil-based adjuvant and administered via the subcutaneous route. This administration route is known to elicit strong humoral and cell-mediated responses (Royo et al. 2025), however, parental vaccines are expected to exert a weaker intestinal mucosal immunity than those delivered through the oral route (Su et al. 2016; Eshraghisamani et al. 2024). In addition, the peripheral immune responses elicited by these parenteral vaccines interfere with both PTB and bTB diagnostic tests (Juste and Perez 2011; Arrazuria et al. 2020). Intestinal mucosal immunity is fundamental for protection against the invasion of most enteric pathogens (Perez-Lopez et al. 2016), including *Map*, although its mechanisms are poorly understood (Facciuolo et al. 2020; Agulló-Ros et al. 2022). Among these mechanisms, differences in the populations of antigen-presenting cell (APCs) involved in each vaccination route induce distinct T cell responses, which could subsequently influence the immune responses established at the infection site (Pasetti et al. 2011; Arrazuria et al. 2020). In a previous study, we compared an oral (OV) and intradermal (IDV) experimental vaccines with a commercial subcutaneous vaccine (SCV) against PTB in goats (Criado et al. 2024). Although the experimental prototypes did not match the protection induced by the SCV, the OV reduced lesion severity, whereas the IDV reduced the tissue bacterial load.

The mechanisms underlying the immune responses elicited by vaccination, as well as the lack of protection observed in some animals, remain poorly understood. Regarding the local response against *Map* infection, macrophages are the most studied immune cells (Delgado et al. 2010; Fernández et al. 2017; Jenvey et al. 2019). Inducible nitric oxide synthase (iNOS), one of the nitric oxide (NO) synthase isoforms is a hallmark molecule of M1 macrophages. It is expressed by a variety of cells after induction by cytokines and other stimuli. Although NO can eliminate some pathogens, it can also damage normal tissue cells, generating pathogenic effects (Xue et al. 2018), and its involvement in the control of mycobacterial infections is still unclear (Yang et al. 2009). PTB infection induces a local increase in its expression (Delgado et al. 2010), and it is highest within focal lesions, particularly in vaccinated animals, and lowest within diffuse multibacillary lesions (Fernández et al. 2017; Zapico et al. 2025). CD163 is associated with M2 macrophages, it is a scavenger receptor for the hemoglobin/haptoglobin complex, which mediates anti-inflammatory and anti-microbial events during bacterial infections (Suzuki et al. 2017). The number of M2 macrophages is higher in the multibacillary lesions of animals with clinical PTB compared to subclinical and non-infected animals, and this macrophage phenotype is considered permissive to intracellular *Map* growth (Fernández et al. 2017; Jenvey et al. 2019). Several studies have addressed the effect of *Map* infection on local cytokine production and expression in cattle (Lee et al. 2001; Coussens et al. 2004; Khalifeh and Stabel 2004; Tanaka et al. 2005; Magombedze et al. 2015; Roussey et al. 2016; Facciuolo et al. 2020) and sheep (Alzuherri et al. 1996; Smeed et al. 2007; Kumar et al. 2010; Sonawane and Tripathi 2016), as can be seen in Additional file 1. In general, this response has been investigated in the ileum and mesenteric lymph nodes from animals with advanced PTB, but the results have often been contradictory. Overall, an upregulation in the expression of both pro- (IFN-γ, TNF, IL-1α, IL-1β, and IL-8) and some anti-inflammatory cytokines (IL-10 and TGF-β) has been reported. Additionally, it has been described that different intestinal regions, particularly jejunal versus ileal Peyer's patches (JPP and IPP), exhibit distinct mucosal cytokine responses against infection (Facciuolo et al. 2020).

The influence of vaccination on local cell populations (e.g. macrophages and lymphocytes) after challenge or infection of ruminants with *Map* has been scarcely analyzed in *in vivo* studies. In cattle vaccinated with Silirum®, a very significant increase in γδ T cells numbers in the JPP was observed 28 days after *Map* challenge, and these cells were also increased in the IPP. In addition, an increase in the number of myeloid cells (CD14^+^) was also observed in both intestinal regions (Facciuolo et al., 2020). At later stages, non-vaccinated naturally infected goats, showed an increase in total macrophage (CD68^+^) numbers and in the M1 (iNOS^+^) subset, when compared with Gudair® vaccinated goats. This increase was associated with a higher bacterial burden (Agulló-Ros et al. 2022).

Traditionally, in the pathogenesis of mycobacterial diseases, greater emphasis has been placed on the study of cellular immunity, while humoral responses have been considered secondary. Yet, recently, an early and robust peripheral humoral response following PTB vaccination has been associated with effective vaccine protection in sheep (Pooley et al. 2019). However, in infected animals, the onset of antibody production is considered indicative of the progression of infection and is associated with diffuse intestinal lesions and clinical disease (Vazquez et al. 2013). Regarding the local humoral response, secretory IgA is the dominant antibody isotype in secretions, constituting an essential component of mucosal immunity (Mach and Pahud 1971; Li et al. 2020). *Map*-infected animals show very low specific IgA levels in sera (Abbas and Riemann 1988), but in the intestinal mucosa, specific IgA is produced in both clinically infected and healthy sheep exposed to *Map* (Begg et al. 2015). In calves exposed to *Map* proteins, specific IgA production takes place primarily in the JPP (Facciuolo et al. 2016). The role of IgA has been studied in human tuberculosis infection (Xiao et al. 2017), vaccination (Wu et al. 2017) and even passive immunization (Reljic et al. 2006). However, its role in PTB pathogenesis and the impact of vaccination on its levels remain poorly studied. Only a recent study on an oral PTB inactivated vaccine tested in rabbits has demonstrated an increase in total (non-specific) serum IgA levels (Ladero-Auñon et al. 2021).

Summarizing, local immune response to *Map*, as well as the impact of vaccination on this response remains poorly studied, particularly in goats. Additionally, the effect of oral and intradermal vaccination on mucosal immunity has not been explored in ruminants. Therefore, the aim of the present work was to analyze the effect vaccination against PTB through the oral, intradermal, and subcutaneous routes —with or without a subsequent *Map* challenge—, in the mucosal immune response of goats. This approach should generate knowledge that may contribute to improvements in currently available vaccines, as well as to the identification of mucosal immune responses associated with protection and vaccine efficacy.

## 2. Materials and methods

### 2.1. Origin of the samples

The current study was performed on 28 distal jejunal Peyer's patch (DJPP) and jejunal content samples obtained from a previous study. The experimental design, including housing, management, vaccination and challenge regimes, as well as vaccines, and challenge inoculum composition have been described in detail (Criado et al. 2024). Briefly, both experimental vaccines contained the same antigen quantity as Gudair®, 12.5 mg of whole-cell chemically inactivated *Map* (strain 316F) per dose. The OV contained 5 mg of the adjuvant Quil A® (InvivoGen, San Diego, CA, United States), was diluted in 10 mL of physiological saline solution, and was administered orally in two doses separated by 14 days. The IDV did not use an adjuvant, was diluted in 0.4 mL of physiological saline solution and was administered as a single intradermal dose. A total of 28, one-month-old goat kids were vaccinated with the oral (*n* = 6) or intradermal (*n* = 6) experimental vaccines, the subcutaneous commercial vaccine Gudair® (*n* = 6) or were left unvaccinated (*n* = 10).

Subsequently, one-month post-vaccination (1 mpv), half of the goats from each of the vaccinated groups (*n* = 3), and seven of the non-vaccinated goats (*n* = 7) were challenged with *Map.* At 11 mpv, the animals were humanely euthanized by deep sedation with xylazine (Xilagesic®, Calier, Barcelona, Spain) and a subsequent intravenous injection of embutramide, mebezonium iodide, and tetracaine hydrochloride (T61®, MSD, Kenilworth, NJ, USA) followed by exsanguination. Then, regulated, orderly, and complete necropsies were performed.

The final groups constituted, after vaccination and challenge, are summarized in Table 1. This previous work evaluated the peripheral immune responses induced by vaccination and *Map* challenge, as well as several efficacy parameters (lesion severity, bacterial burden in tissues and fecal loads) (Criado et al. 2024). To draw conclusions about the effects of vaccination on the local immune response studied here, some of the data obtained in the previous work were reused and compared. The lesion classification for the DJPP sections was established in a blinded manner with respect to the experimental group, following previously described criteria (González et al. 2005; Criado et al. 2024), and the lesions were classified as: focal, when granulomas were restricted to the lymphoid tissue of the PP; multifocal, when small granulomas were also located in the lamina propria (LP); and diffuse when granulomas were widespread throughout the intestinal mucosa. No lesions were observed in the non-challenged animals.

**Table 1.** Experimental groups established. Number of animals per category, and group abbreviation. Individual data obtained in the previous experiment (Criado et al. 2024) and used for the FAMD and correlation analyses.

Experimental group	Animal	Type of lesion (DJPP)	*Map* DNA load (fg/g)		Granuloma counts		Peripheral responses^a^		Infection status	Type of lesion^b^
			DJPP	Feces (10 MPI)	DJ	DJPP	IGRA	IgG		
No challenge Oral vaccine (OV, *n* = 3)	11	No Lesion	0	0	0	0	1.49	−0.02	Not infected, No lesion (NO-INF, *n =* 12)	
	12	No Lesion	0	0	0	0	1.07	−0.04		
	13	No Lesion	0	0	0	0	1.05	−0.05		
No challenge Intradermal vaccine (IDV, *n* = 3)	31	No Lesion	0	0	0	0	1.57	−0.05		
	32	No Lesion	0	0	0	0	0.83	−0.04		
	33	No Lesion	0	0	0	0	1.38	−0.05		
No challenge Subcutaneous vaccine (SCV, *n* = 3)	51	No Lesion	0	0	0	0	2.25	2.05		
	52	No Lesion	0	0	0	0	2.89	2.11		
	53	No Lesion	0	0	0	0	3.47	2.10		
No challenge No vaccine (NV, *n* = 3)	71	No Lesion	0	0	0	0	1.31	0.52		
	72	No Lesion	0	0	0	0	0.90	−0.01		
	73	No Lesion	0	0	0	0	1.01	−0.03		
Challenge Oral vaccine (OV-INF, *n* = 3)	21	Focal	1.56	1.15	0	2	1.20	−0.05	Infected (INF, *n =* 16)	Focal (F, *n* = 5) Multifocal (MF, *n* = 3) Diffuse (D, *n* = 8)
	22	Multifocal	325.11	29.95	10	3	3.23	4.60		
	23	Diffuse	1964.79	4867.71	130	30	3.85	8.31		
Challenge Intradermal vaccine (IDV-INF, *n* = 3)	41	Diffuse	771.38	66.33	6	94	5.13	0.24		
	42	Diffuse	392.30	2200.43	205	66	4.56	5.15		
	43	Focal	0.00	6.80	6	13	2.33	0.27		
Challenge subcutaneous Vaccine (SCV-INF, *n* = 3)	61	Focal	0.50	1.46	29	16	4.10	4.64		
	62	Focal	0.62	0.00	0	1	3.56	1.88		
	63	Multifocal	105.58	51.87	14	50	2.64	4.68		
Challenge No vaccine (NV-INF, *n* = 7)	81	Multifocal	2.86	5.79	1	32	1.36	1.84		
	82	Diffuse	246.72	83.41	1	220	3.99	1.83		
	83	Diffuse	326.17	80.06	56	250	3.86	4.64		
	84	Diffuse	23.20	11.29	20	70	2.87	3.48		
	85	Diffuse	889.58	84.66	32	130	3.75	1.94		
	86	Focal	0.00	0.00	0	2	0.78	−0.03		
	87	Diffuse	2793.89	7.60	12	100	2.70	4.57		

^a^ IgG levels by 10 MPI, expressed as the log_2_ (OD_450_ index) or log_2_ (OD_450_ ratio).

^b^ The lesional classification of the challenged animals was assigned based on the lesion observed at the distal jejunal Peyer's patch (DJPP) sections. DJ: distal jejunum. IGRA: results of the IFN-γ release assay, performed in response to *M. avium* purified protein derivative (aPPD).

### 2.2. Sampling

Jejunal content samples were collected in sterile tubes (50 mL centrifuge tubes, Falcon™, Corning, NY, USA) and frozen at −80 °C. Also, at least 5 cm of a DJPP was collected, thoroughly washed in sterile PBS, and kept at 4 °C in RPMI1640 medium with GlutaMax™ (61870010, Gibco®, Paisley, United Kingdom), supplemented with 10% fetal bovine serum (10500064, Gibco®, Paisley, United Kingdom), 1% HyClone™ Antibiotic/Antimycotic solution (Cytiva, Washington DC, WA, USA) and 50 uM of 2-β-mercaptoethanol (Gibco®, Paisley, United Kingdom) until further processing (see Section 2.4).

DJPP tissue samples were selected for (i) immunohistochemical and immunofluorescence analysis, (ii) mRNA transcription analysis, and (iii) quantification of the IFN-γ and IL-4 production by tissue-isolated mononuclear leukocytes, as this tissue showed the highest numbers of lesions and *Map* loads (Criado et al. 2024), with a median of 41 granulomas per section/area, and 176.14 fg of *Map* DNA per g of tissue, in challenged animals (Table 1). For mRNA expression analysis, the excess tissue surrounding the PP was removed, and the mucosa was scraped, minced, and frozen at −80 °C in RNAlater (Invitrogen™, Carlsbad, CA, USA). Additionally, formalin-fixed, paraffin-embedded (FFPE) tissue samples, conventionally processed as described (Criado et al. 2024), were used for immunohistochemical and immunofluorescence analysis.

### 2.3. Immunohistochemistry

The immunohistochemical staining was performed as previously described (Criado et al. 2024), using the Agilent EnVision™ Peroxidase/DAB detection system (Agilent Technologies, Santa Clara, USA). Primary antibodies and epitope unmasking conditions are provided in Additional file 2*.* For the analysis of the immunohistochemical staining, a differential count was carried out in the lamina propria (LP) and gut-associated lymphoid tissue (GALT), given that they constitute immunologically different compartments. In each DJPP section, 10 random fields at 200× magnification (equivalent to 1.42 mm^2^) from each area (LP and GALT) were analyzed as previously described (Criado et al. 2024). Counts where performed using the Nikon® Eclipse E600 microscope fitted with a Nikon® DS-Fi1 digital camera (Nikon, Tokyo, Japan). Immunolabeled cells were counted using the NIS-Elements v3.2 (Nikon, Tokyo, Japan) counting tool, and the data was expressed as the mean number of labeled cells per mm^2^. All counts were performed in a blinded manner with respect to the experimental group. Micrographs were taken using the Nikon® Eclipse Ci-L, coupled with the Nikon® DS-Fi3 camera (Nikon, Tokyo, Japan).

### 2.4. Immunofluorescence staining of tissue sections

In order to investigate whether WC1 γδ T lymphocytes could contribute to local IFN-γ production, and if the local IgA production was related with the presence of *Map,* two double immunofluorescence assays were performed. For the immunofluorescence assays, representative FFPE DJPP samples including different pathological and immunological scenarios were selected: (i) a control animal (goat 71); (ii) an animal with focal lesions, a low bacterial load and a high number of WC1 γδ T (goat 62); and (iii) two animals with diffuse lesions and a high bacterial load, where *Map* had been detected through IHC (goats 83 and 87).

The immunofluorescence staining was performed on 3 µm-thick tissue sections, which were placed onto poly-L-lysine-coated slides (SuperFrost™ Plus Adhesion slides, Thermo Fisher Scientific, Waltham, USA). Heat-induced epitope retrieval (HIER), deparaffination and hydration were performed using PT-Link and Dako Target Retrieval Solutions (Agilent Technologies, Santa Clara, CA, USA), as specified in Additional file 3. Afterwards, three 5 min washes (Wash buffer, Agilent Technologies, Santa Clara, USA) were performed, and to reduce background fluorescence, the sections were immersed in 3% H_2_O_2_ in methanol for 30 min in darkness at room temperature. After washing twice, permeabilization and blocking were performed with 0.25% Triton™ X-100 (Sigma-Aldrich, Darmstadt, Germany) and 3% bovine serum albumin (BSA) (Roche Diagnostics, Mannheim, Germany) in PBS for 1 h at 37 °C. After being washed twice, the sections were incubated overnight at 4 °C in a humidified chamber with primary antibodies against either *Map* or WC1 (see Additional file 3). Animal-free blocker® and diluent R.T.U. (Vector Laboratories, CA, USA) was used for all the antibody dilutions.

After three washes, the sections were incubated for 1 h at room temperature with Goat Anti-Rabbit IgG H&L conjugated to Alexa Fluor® 488 (Invitrogen™, Carlsbad, CA, USA) at 4 µg/mL. Following two washes, a second incubation was performed overnight with primary antibodies against either IgA or IFN-γ conjugated to Alexa Fluor® 647, using the Alexa Fluor® 647 Conjugation Kit (Fast) – Lightning-Link® (Abcam, Cambridge, UK) (Additional file 3), following the manufacturer's instructions. The Vector® TrueVIEW® Autofluorescence Quenching Kit (Vector Laboratories, CA, USA) was used to reduce tissue autofluorescence and, after two washes, the slides were stained using DAPI (4′,6-diamidino-2-phenylindol) (Invitrogen™, Carlsbad, CA, USA) at a concentration of 2.5 μg/mL. Micrographs were taken using the direct microscope Eclipse Ni-E (Nikon, Tokyo, Japan) equipped with the Prime BSI Scientific CMOS scientific camera (Photometrics® Prime BSI™, Scottsdale, AZ, USA).

### 2.5. RNA extraction, reverse transcription, and quantitative real-time PCR

Total RNA was extracted from 20 mg of DJPP using the Maxwell® RSC simplyRNA Tissue Kit (Promega, WI, USA) with the Maxwell 16 Instrument (Promega, Madison, WI, USA) following the manufacturer's instructions. The RNA was quantified using a QuantiFluor™ RNA System Kit and Quantus™ Fluorimeter (Promega, Madison, WI, USA) following the manufacturer's instructions, and the RNA purity was assessed using a NanoDrop 1000 (Thermo Fisher Scientific, Waltham, USA), with the acceptable 260/280 absorbance ratio set to 1.7. RNA integrity was checked assessing the integrity of the 18S and 28S ribosomal subunits, through electrophoresis using a 1% agarose gel with GelRed® nucleic acid gel stain (Biotium, CA, USA). Then, reverse transcription to cDNA of up to a total of 2500 ng of RNA was performed using SuperScript™ VILO™ Master Mix (Invitrogen™, Carlsbad, CA, USA) and the SimpliAmp™ Termal Cycler (Applied Biosystems™, Warrington, UK) (Gutiérrez-Expósito et al. 2020). The obtained cDNA was diluted in nuclease-free water to 10 ng/μL for its use in quantitative PCR real-time (qPCR) assays.

PCR reactions were performed in a 96-well plate (Applied Biosystems™, Warrington, UK) using 10 µL of PowerUp™, SYBR™ Green master mix (Applied Biosystems™, CA, USA), 10 µM of each primer and 2 µL of the diluted cDNA samples on a 7500 Fast Real-Time PCR System (Applied Biosystems™, CA, USA). Amplification efficiencies were analyzed including a seven-point standard curve for each target gene on every plate, which was prepared from 10-fold serial dilutions of a starting concentration of 1 ng/µL of a conventionally prepared PCR product. All cDNA samples were prepared in parallel and analyzed at the same time.

Primer sequences for TNF, IFN-γ, IL-1β, IL-4, IL-10, IL-17A, iNOS and the reference genes β-actin, and glyceraldehyde 3-phosphate dehydrogenase (GADPH) have been previously described (Additional file 4) (Peletto et al. 2011; Arranz-Solís et al. 2016; Arteche-Villasol et al. 2021). The relative quantification of the mRNA expression levels (fold change in expression) was carried out using the 2^−ΔΔCt^ relative quantification method as previously described (Livak and Schmittgen 2001). Briefly, to assess the changes in mRNA expression, for each sample and gene of interest, the ΔCt value (gene of interest Ct – mean Ct of the reference genes) was calculated. Then, the mean ΔCt value from the NV group animals was used as a calibrator to calculate the ΔΔCt (problem sample ΔCt—mean ΔCt of NV animals) of each transcript. The fold change (FC) in gene expression was calculated (2^−ΔΔCt^), and a log transformation (log_2_FC) was performed (Criado et al. 2023).

### 2.6. Tissue mononuclear leukocytes isolation and determination of the *ex vivo* IFN-γ and IL-4 production after aPPD stimulation

Tissue mononuclear leukocytes were isolated as previously described (Arteche-Villasol et al. 2022). The excess tissue around each DJPP was removed, and the mucosa was scraped and minced using a scalpel blade. The tissue was then suspended in 11 mL of PBS with 2 mM EDTA and processed with a stomacher blender (Masticator, IUL Instruments, Barcelona, Spain) for 2.5 min. Then, 10 mL from the upper homogenized portion was passed through a 40-μm Falcon™ cell strainer (Thermo Fisher Scientific, Waltham, USA), and the resultant suspension was layered over an equal volume of Lymphoprep™ (STEMCELL Technologies®, Grenoble, France) and centrifuged at 800 g for 30 min. The cells from the interface layer were taken and washed three times with PBS supplemented with 2 mM EDTA, counted in a Neubauer chamber, and resuspended at a final concentration of 2 × 10^6^ cells × mL^−1^ in RPMI1640 supplemented as mentioned above. The cell viability determined by trypan blue dye exclusion was >90%.

For the study of the local cell-mediated immune response to re-stimulation, after isolation, 3 × 10^6^ cells were seeded per well (24-well plates) in 1.5 mL of supplemented RPMI1640. Then, 100 μL of sterile phosphate buffered saline (PBS) or *M. avium* purified protein derivative (aPPD) (CZ Veterinaria, Porriño, Spain) at a final concentration of 20 μg/mL were added to each well in duplicates. After incubation (20 h at 37 °C, 5% CO_2_), the plates were centrifuged at 750 *g* for 15 min, and the cell culture supernatants were collected and stored at −20 °C.

Then, the assay for the determination of IFN-γ, BOVIGAM® TB Kit (Thermo Fisher Scientific, Waltham, MA, USA), was used to measure the IFN-γ concentration in the supernatant samples. The assay was performed in duplicate following the manufacturer's instructions and interpreted as previously described (Delgado et al. 2012). Results were expressed as the ratio of the mean optical density, measured spectrophotometrically at a wavelength of 450 nm (OD_450_), of the aPPD-stimulated cell supernatants, and the mean OD_450_ of the supernatants from the nil-stimulated (PBS) cells. Then, the values were logarithmically transformed [log_2_(x + 1)].

The levels of IL-4 were measured using the Bovine IL-4 ELISA^BASIC^ kit (3118-1H, Mabtech, AB, Sweden) following the manufacturer instructions. The assay was performed in duplicate, and the plates were read at 450 nm. The IL-4 concentration in the supernatants was determined using the standard curve provided in the kit. This curve was generated by plotting the OD_450_ values against known concentrations of recombinant IL-4, enabling the interpolation of IL-4 concentrations in the samples. The results were then expressed as the difference between the IL-4 concentration measured in the aPPD-stimulated cell supernatants and the supernatants from the nil-stimulated (PBS) cells.

### 2.7. Determination of intestinal content IgA

The jejunal content samples were thawed and homogenized, then 2 g were resuspended in 4 mL of PBS, 20 mM EDTA and 2 mM phenylmethylsulfonyl fluoride (PMSF, Thermo Fisher Scientific, Waltham, MA, USA), and centrifuged (300 *g*, 10 min, 4 °C). Afterwards, 2 mL of the supernatant were filtered and passed through a 0.2 μm filter.

Then the ID Screen® *Paratuberculosis* Indirect (IDvet, Gabrels, France) ELISA test was modified to measure the *Map*-specific IgA in the intestinal content. The ELISA was performed following the manufacturer's instructions, with overnight incubation and two modifications: (i) Six, instead of three washes were performed in every wash step; (ii) The HRP-conjugated, anti-ruminant IgG included in the kit was substituted with the HRP-conjugated Rabbit anti-Goat IgA antibody (Reference: AAI44P, Bio-Rad laboratories Inc., Hercules, CA, USA) at a 1:20,000 dilution.

The optical density was measured spectrophotometrically at a wavelength of 450 nm, and the results were expressed as a ratio between the mean OD_450_ of each sample duplicates and the mean OD_450_ of the animals from the NV group. The results were then logarithmically transformed [log_2_(x + 1)].

### 2.8. Statistical analysis

Given the large amount of data and variables, a factor analysis of mixed data (FAMD) was conducted to analyze the relationships between quantitative and qualitative variables. The dataset included a total of 24 variables (Table 2): 4 qualitative (experimental group—OV, IDV, SCV, NV, OV-INF, IDV-INF, SCV-INF, NV-INF—, vaccine administered, infection status and lesion classification), and 20 quantitative variables (IHC cell counts, relative mRNA expression of cytokines, cytokine production by aPPD-stimulated mononuclear leukocytes and IgA in jejunal contents). The analysis focused on understanding the relationships among these variables across the different experimental groups. Additionally, in order to analyze the relationships between local and peripheral immune responses and infection severity by the endpoint of the experiment, a second FAMD was conducted, including some of the results obtained in our previous work (Criado et al. 2024). This ‘extended’ FAMD incorporated six additional variables (Table 2): peripheral humoral and cellular immune responses at 10 MPI, granuloma counts, and *Map* DNA load in tissues and feces at 10 MPI.

**Table 2.** Variables included in the factorial analysis of mixed data (FAMD).

Type of variable			Variable (abbreviation)
Qualitative variables	Experimental group (group)		
	Vaccine administered (vaccine)		
	Infection status (infection)		
	Lesion classification in the DJPP/DJ (Lesion.Classification)		
Quantitative variables		IHC cell counts in DJPP (No. of labeled cells/mm^2^)	Iba1^+^ cells in the LP (Iba1.LP)
			Iba1^+^ cells in the GALT (Iba1.GALT)
			iNOS^+^ cells in the LP (iNOS.LP)
			iNOS^+^ cells in the GALT (iNOS.GALT)
			CD163^+^ cells in the LP (CD163.LP)
			CD163^+^ cells in the GALT (CD163.GALT)
			WC1^+^ cells in the LP (WC1.LP)
			WC1^+^ cells in the GALT (WC1.GALT)
			IgA^+^ cells in the LP (IgACells.LP)
			IgA^+^ cells in the GALT (IgACells.GALT)
		Relative mRNA cytokine expression in the DJPP (log_2_FC)	IFN-γ (IFN.Transcription)
			IL-4 (IL4.Transcription)
			IL-10 (IL10.Transcription)
			IL-1β (IL1β.Transcription)
			TNF (TNF.Transcription)
			IL-17A (IL-17A.Transcription)
			iNOS (iNOS.Transcription)
		Local, specific or recall immune responses against *Map* (ELISA)	IFN-γ production by aPPD-stimulated DJPP mononuclear leukocytes (Recall.IFN)
			IL-4 production by aPPD-stimulated DJPP mononuclear leukocytes (Recall.IL4)
			Anti-*Map* IgA in the jejunal content (IgA.Jejunum)
		*Map* DNA loads (fg/g)*	In the DJPP (Map.PCR.DJPP)
			In feces, by 10 MPI (Map.PCR.Fecal)
		Granuloma counts (per section/area)*	In the LP of the DJ (Granuloma.J)
			In the GALT of the DJPP (Granuloma.DJPP)
		Peripheral immune responses by 10 MPI*	IFN-γ release assay in peripheral blood in response to aPPD (IFN.Blood)
			Anti*-Map* IgG in blood serum (IgG.Serum)

^*^ Asterisks indicate data collected in our previous work (Criado et al. 2024), which is included in the extended FAMD (Figure 1) but not in the local FAMD (Additional File 5). DJPP (Distal jejunal Peyer's patch), DJ (Distal jejunum), LP (Lamina propria), GALT (Gut-associated lymphoid tissue), MPI (Months post-infection), aPPD (*M. avium* purified protein derivative).

**Figure 1.**
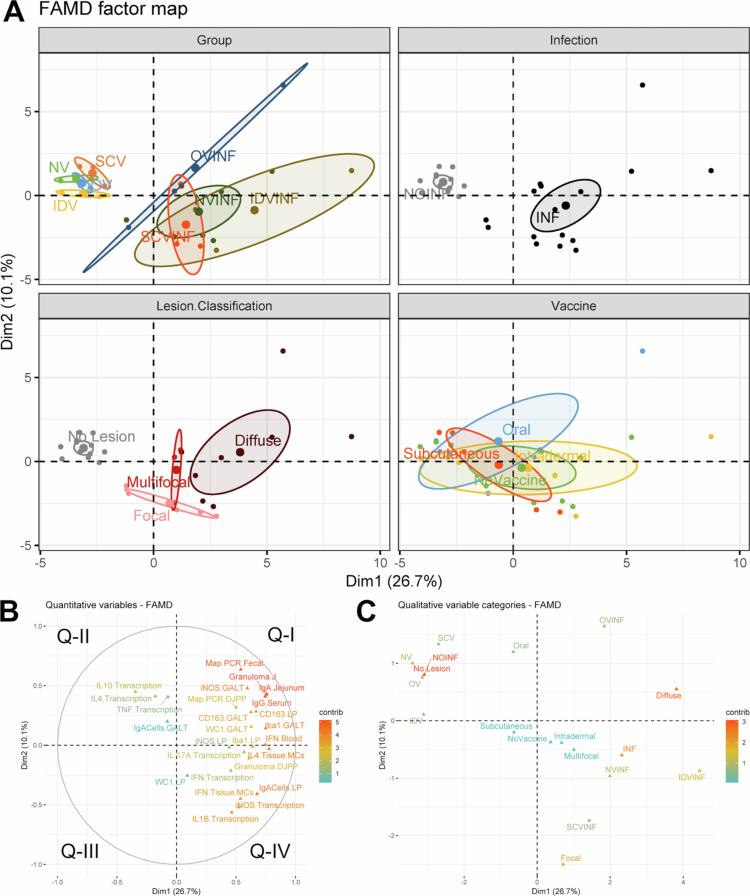
Extended factorial analysis of mixed data (FAMD), including immune response and infection severity parameters. (A) Elliptical factor map, individuals are indicated with a dot, colored based on their experimental group, infection status, lesion classification or vaccine received. The ellipses are drawn around the centroid (mean position) of each group and capture the spread and variability of the data points within that group. Its size reflects the concentration of the data; larger ellipses indicate greater variability among individuals in that group. (B) Quantitative variables contribution plot. Q (quadrants). (C) Qualitative variables contribution plot. Abbreviations for the groups are provided in Table 1, and for the variables in Table 2.

The quantitative variables were standardized (centered and scaled) using the formula: *Z* *i* = (*X* *i*—*μ*)/*σ*, where *Z* *i* is the standardized value, *X* *i* ​is the original value, *μ* is the mean of the variable, and *σ* is the standard deviation. This standardization process ensures that all quantitative variables have a mean of 0 and a standard deviation of 1, allowing for a fair comparison of mixed data types in the FAMD.

For further statistical analysis, non-parametric tests were employed to assess the data categorized by experimental groups or lesion types, given that some groups consisted of only three animals. *Ad hoc* comparisons among the experimental groups (between non-challenged groups, challenged groups, and each non-challenged group and its challenged counterpart) were performed using two-sided Mann–Whitney U tests. The remaining comparisons among the experimental groups, as well as those between lesion types, were assessed using the Kruskal‒Wallis test, followed by Dunn's *post hoc* test. To account for multiple comparisons and control the false discovery rate, the Benjamini‒Hochberg correction was applied during significance testing when comparing experimental groups.

To analyze the differences when grouping animals by their infection status (non-infected vs infected), the Shapiro‒Wilk test was first applied to assess the normality of each variable. Based on the results, appropriate parametric (*t*-test) or non-parametric (Mann–Whitney U test) analyses were conducted to compare the two groups.

The Spearman rank test with Benjamini‒Hochberg correction was performed to assess the correlation between the local expression of the different cytokines studied, between the local and peripheral *Map*-specific immune responses and between IgA levels in the intestinal contents and both the *Map* load in feces and DJPP.

All the statistical analyses were performed with R software version 4.2.3 (R Core Team 2022). The following R packages were employed for the statistical analysis: ‘factoextra’ (v1.0.7), ‘FactoMineR’ (v2.11), ‘ggforce’ (v0.4.2), ‘ggplot2’ (v3.4.2), ‘plotly’ (v4.10.4), ‘psych’ (v2.4.3), ‘corrplot’ (v0.92), ‘rstatix’ (v0.7.2), ‘ggpubr’ (v0.6.0.999), ‘lubridate’ (v1.9.2), ‘forcats’ (v1.0.0), ‘stringr’ (v1.5.0), ‘dplyr’ (v1.1.1), ‘purrr’ (v1.0.1), ‘readr’ (v2.1.4), ‘tidyr’ (v1.3.0), ‘tibble’ (v3.2.1), and ‘tidyverse’ (v2.0.0).

## 3. Results

To facilitate interpretation of the multidimensional dataset, a global multivariate analysis was first used to identify the main sources of variation and to support subsequent grouping of animals according to lesion type. Then, results are presented in the following sections according to a biological rationale: counts of myeloid and innate-like lymphoid cell populations (macrophages and WC1 + γδ T cells), and IgA plasma cells, cytokine expression profiling, and finally *Map*-specific responses, including recall cytokine responses to aPPD (IFN-γ and IL-4 production), and anti-*Map* IgA production.

### 3.1. Factorial analysis revealed that infection and lesion type, rather than the vaccine administered, are the predominant factors influencing the variability observed in the immune responses

The extended FAMD, including data on peripheral immune responses, granuloma counts and *Map* loads, from our previous work (Criado et al. 2024), can be seen in Figure 1. The results from the FAMD, including only parameters from the local immune responses, are presented in Additional file 5. In both analyses, individuals were grouped in a relatively similar manner in the factor maps (Additional file 5A and Figure 1A). Additionally, both FAMDs explained a similar percentage of variance, with the first two principal components accounting for 34.6% of the variance, including only local immune responses, and 36.8% in the extended FAMD. To provide a broader perspective and establish relationships between local and peripheral immune responses, as well as bacterial burden, the analysis of the results will focus on the extended FAMD (Figure 1).

When analyzing the extended FAMD factor map (Figure 1A), neither the vaccine employed (or lack thereof) nor the experimental group (which integrates both vaccination and infection status) resulted in a clear separation of the groups. This indicates that these factors contribute minimally to the differentiation of animals within the multivariate space. In contrast, when the grouping was conducted based on the infection status, it resulted in better clustering. In this regard, non-challenged animals are clustered very tightly, whereas challenged animals display more variability. Additionally, within challenged animals, clustering was observed when grouping by lesion type (Figure 1A). Overall, these findings indicate that infection status and lesion type, rather than vaccination, are the primary contributors influencing the local immune response parameters studied. This is further substantiated by the analysis of the qualitative variables, which reveals that categories regarding infection status (infected and non-infected), and lesion severity (diffuse, focal and no lesion) are the five with the most significant contributions (Figure 1C). Because of this, throughout the next sections, the individual results will be analyzed by grouping the animals not only by experimental group but also by infection status and lesion type.

When evaluating the contribution of the quantitative variables to the overall variance (Figure 1B), most exhibit a comparable degree of influence on both dimensions 1 (Dim1, horizontal) and 2 (Dim2, vertical). This highlights the complex interplay between the immune response, infection, and lesion severity. Therefore, a quadrant-based analysis was performed, which should provide insights into the relationships among variables. Quadrant I (Q-I, upper right), which includes variables that have a high positive contribution to both Dim1 and Dim2, encompasses increases in variables somewhat related with infection severity: *Map* load in feces and DJPP, granuloma counts in jejunal LP and both the local (IgA) and peripheral (IgG) humoral immune responses. Also, regarding the local response, increases in the numbers of M2 macrophages in LP, and all the cell populations studied in GALT, fell within this quadrant.

Quadrant II (Q-II, upper left) (Figure 1B) includes variables that have a high positive contribution to Dim2 and a high negative contribution to Dim1. A correlation was observed among the local expressions of IL-10, IL-4, and TNF, which appeared to be negatively associated with the variables included in quadrant IV (Q-IV, lower right). This quadrant includes variables that have a high negative contribution to Dim2 and a high positive contribution to Dim1; IL-1 β and IFN-γ expression, recallIFN-γ response and WC1^+^ γδ T cells numbers in the LP. Finally, some variables which mainly contributed to Dim1 were also situated within this quadrant: IL-17A expression, IL-4 recall response, the IGRA results (IFN-γ release assay, performed in peripheral blood, in response to aPPD), as well as total (Iba1^+^) and M1 (iNOS^+^) macrophages in the LP.

### 3.2. Macrophage subsets distribution and numbers in the intestinal mucosa

**Figure 2.**
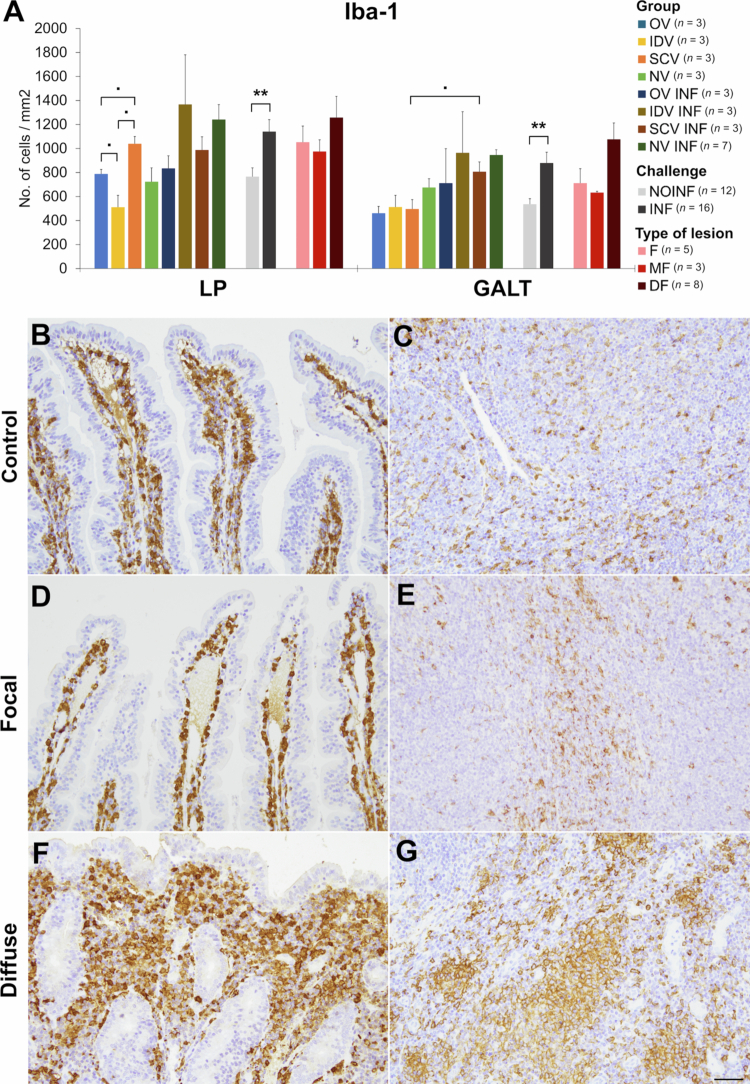
Numbers and distribution of the macrophages (Iba1^+^cells) in the distal jejunum Peyer's patches. (A) Mean cell counts of the immunolabeled cells present in the mucosa. All values are expressed as means, and error bars represent the standard error. Brackets indicate statistically significant differences between groups. The dots indicate a *p* ≤ 0.1; **p < 0.01. Photomicrographs of representative sections from the lamina propria (LP) (B, D, F) and gut-associated lymphoid tissue (GALT) (C, E, G). LP subepithelial macrophages and macrophages located in the interfollicular regions of the GALT can be observed in (B, C) control animals and in (D, E) those with focal lesions. In (F, G) animals with diffuse lesions, a large number of macrophages, compose the diffuse granulomatous infiltrates. Abbreviations for the different groups are indicated in Table 1. All images were acquired at the same magnification (200×). Scale bar = 50 µm.

**Figure 3.**
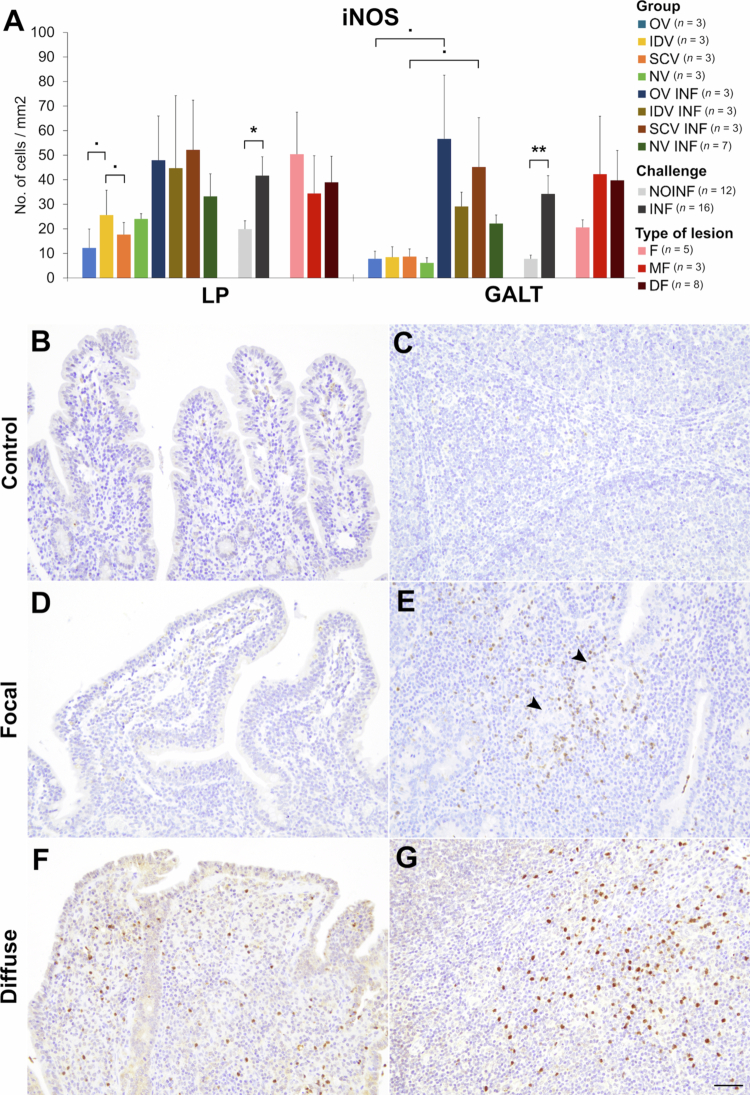
Numbers and distribution of the M1 macrophages (iNOS^+^ cells) in the distal jejunum Peyer's patches. (A) Mean cell counts of the immunolabeled cells present in the mucosa. All values are expressed as means and error bars represent the standard error. Brackets indicate statistically significant differences between groups. The dots indicate a *p* ≤ 0.1; *p < 0.05; **p < 0.01. Photomicrographs of representative sections from the lamina propria (LP) (B, D, F) and gut-associated lymphoid tissue (GALT) (C, E, G). A low number of iNOS+ cells could be observed in (B, C) control animals and, in (D, E) those with focal lesions, where they were only abundant in the periphery of focal granulomas (arrowheads). In (F, G) animals with diffuse lesions, iNOS+ macrophages where abundant among the granulomatous infiltrates. Abbreviations for the different groups are indicated in Table 1. All images were acquired at the same magnification (200×). Scale bar = 50 µm.

**Figure 4.**
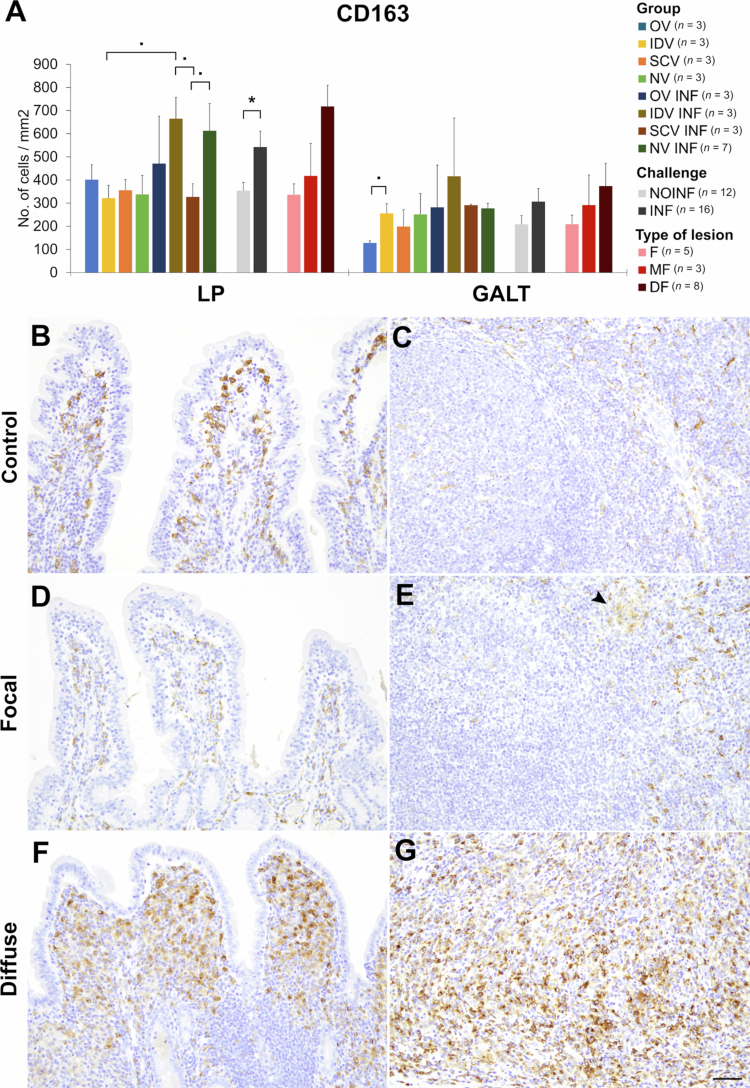
Numbers and distribution of the M2 macrophages (CD163^+^ cells) in the distal jejunum Peyer's patches. (A) Mean cell counts of the immunolabeled cells present in the mucosa. All values are expressed as means and error bars represent the standard error. Brackets indicate statistically significant differences between groups. The dots indicate a *p* ≤ 0.1, *p < 0.05, ***p < 0.001. Photomicrographs of representative sections from the lamina propria (LP) (B, D, F) and gut-associated lymphoid tissue (GALT) (C, E, G). Moderate numbers of LP subepithelial macrophages and of those located in the interfollicular regions of the GALT were CD163^+^ in both (B, C) control animals and (D, E) those with focal lesions. In these, macrophages composing granulomas (arrowheads) were weakly labeled. In (F, G) animals with diffuse lesions, a large number of CD163^+^ macrophages compose the granulomatous infiltrates. Abbreviations for the different groups are indicated in Table 1. All images were acquired at the same magnification (200×). Scale bar = 50 µm.

Results on the quantification of total macrophages (Iba1^+^) and its M1 (iNOS^+^) and M2 (CD163^+^) subsets in the LP and GALT, as well as micrographs illustrating their distribution in those areas, can be seen in Figures 2–4, respectively. No significant differences were observed in the numbers of immune cells within the mucosa across the experimental groups. However, infection status and lesion type significantly influenced several of the markers studied. Consequently, micrographs were taken from representative sections of the animals without lesions (not infected), with focal lesions, and with diffuse lesions in the DJPP.

The number of macrophages (Iba1^+^) was increased in both the LP and GALT in all the challenged groups with respect to their non-challenged counterparts. In both regions, this increase was only significant (*p* < 0.01) when grouped by infection. Moreover, among infected animals, those with diffuse lesions showed the highest macrophage counts, with the increase being significant in the GALT (*p* < 0.01) (Figure 2A). In animals without lesions and with focal and multifocal lesions, Iba1^+^ macrophages were distributed predominantly throughout the subepithelial space of the LP (Figure 2B and D), whereas in the GALT they were mainly located in the interfollicular areas (Figure 2C and E). On the other hand, in the diffuse lesions, large numbers of macrophages infiltrated extensive areas of the LP —leading to the fusion and blunting of villi (Figure 2F)—, and the interfollicular areas of the GALT (Figure 2G).

M1 macrophages (iNOS^+^) were also in higher numbers in the challenged groups in both the LP and GALT. These differences were only significant when grouping all the challenged animals (LP: *p* < 0.05*;* GALT*: p* < 0.01), but in the GALT of the OV-INF and SCV-INF animals, a trend was observed when compared with their non-challenged counterparts (*p* ≤ 0.1). Regarding lesion type, this subset was more abundant in the LP of animals with focal lesions, and in the GALT of those with multifocal and diffuse lesions, in the latter this increase was significant with respect to that of the animals without lesions (*p* < 0.01) (Figure 3A). In terms of distribution, in control animals, moderate amounts of lightly labeled iNOS^+^ macrophages could be seen throughout the subepithelial space of the LP, and they were very scarce in the interfollicular areas of the GALT. However, in the animals with lesions, the distribution was irregular, and strongly labeled macrophages were present in relation to the granulomatous lesions (Figure 3E–G), but not in the areas without lesions, such as the LP of the animals with focal lesions (Figure 3D).

Similarly, M2-polarized macrophages (CD163^+^) were in higher numbers in all the challenged groups (Figure 4A), except in the LP of the SCV-INF group, which exhibited numbers comparable to its not challenged counterpart (SCV). The number of CD163 macrophages was particularly influenced by lesion type, with a progressive increase in both compartments (LP and GALT) in more severe lesions. This increase was significant in the LP of the animals with diffuse lesions with respect to both the animals without lesions and those with focal lesions (*p* < 0.001). In terms of distribution, control animals and those with focal lesions exhibited a similar localization of M2 macrophages; however, these macrophages were present in fewer numbers compared to the total macrophage population (Figure 4A–D​​​​​). Also, in the animals with focal lesions, the epithelioid macrophages forming the granulomas present in the interfollicular areas of the GALT were generally CD163^+^, although they showed only weak labeling (Figure 4E). In contrast, in the animals with diffuse lesions, the macrophages composing the granulomatous infiltrates were always intensely labeled with CD163^+^ (Figure 4F and G).

### 3.3. WC1 γδ T lymphocytes distribution and numbers in the intestinal mucosa

**Figure 5.**
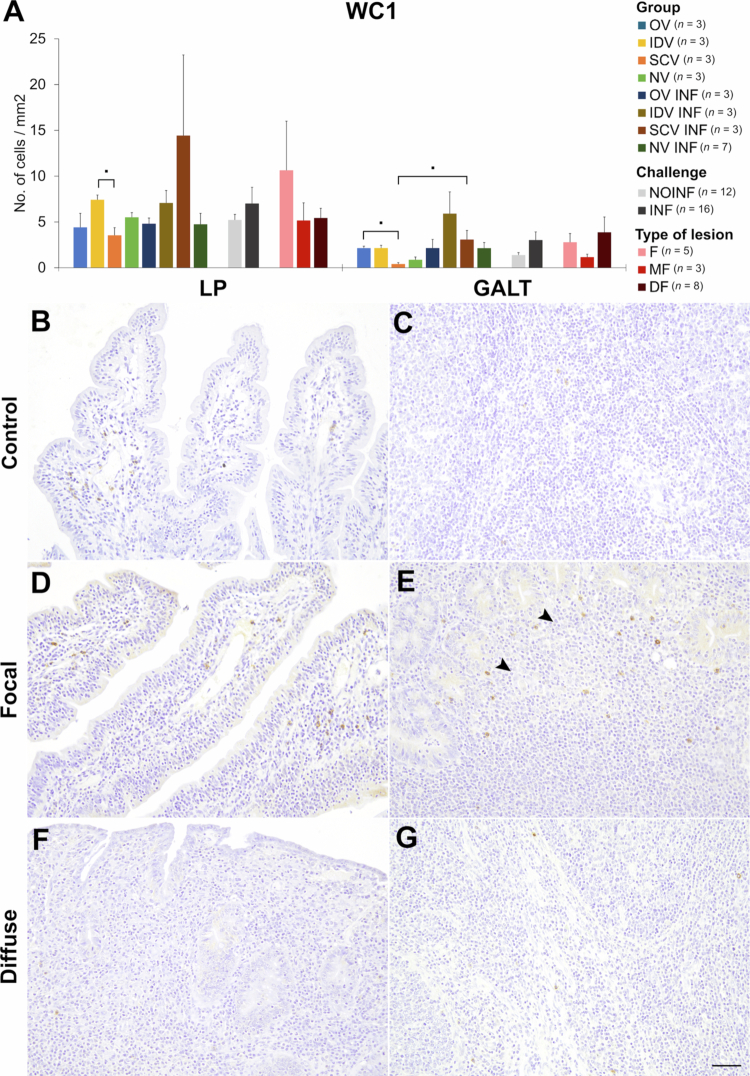
Numbers and distribution of the WC1 γδ T lymphocytes (WC1^+^ cells) in the distal jejunum Peyer's patches. (A) Mean cell counts of the immunolabeled cells present in the mucosa. All values are expressed as means and error bars represent the standard error. Brackets indicate statistically significant differences between groups. The dots indicate a *p* ≤ 0.1. Photomicrographs of representative sections from the lamina propria (LP) (B, D, F) and gut-associated lymphoid tissue (GALT) (c, e, g). (B, C) Control animals showed low numbers of WC1^+^ cells. (D, E) WC1+ cells were more abundant in some areas of the LP of those with focal lesions, but were particularly abundant in relation with focal granulomas (arrowheads) within the interfollicular areas of the GALT. (F, G) Animals with diffuse lesions showed low numbers of WC1^+^ cells. Abbreviations for the different groups are indicated in Table 1. All images were acquired at the same magnification (200×). Scale bar = 50 µm.

There were no clear differences in the WC1 γδ T lymphocytes counts between the groups, and its distribution throughout the mucosa was very variable among the challenged individuals, with no significant differences detected between the experimental groups, infection status or lesion type (Figure 5). However, the mean counts were higher in infected individuals, particularly in the LP of animals with focal lesions and in the GALT of those with diffuse lesions (Figure 5A). Regarding its distribution, they were preferentially located in the subepithelial space of the LP of control animals and were scarce in the GALT (Figure 5B and C). In the sections with focal lesions, the distribution was similar in the LP (Figure 5D), but they were more abundant in the interfollicular areas of the GALT, particularly when close to the LP and in relation to the focal granulomas, which sometimes were surrounded by positively immunolabeled lymphocytes (Figure 5E). In animals with diffuse lesions, their distribution was very irregular, sometimes they were in close relationship with the granulomatous infiltrates, whereas in other cases, they were completely absent (Figure 5F). In the GALT they were regularly distributed throughout the interfollicular areas, similarly to that seen in control animals, but in slightly higher numbers (Figure 5G).

**Figure 6.**
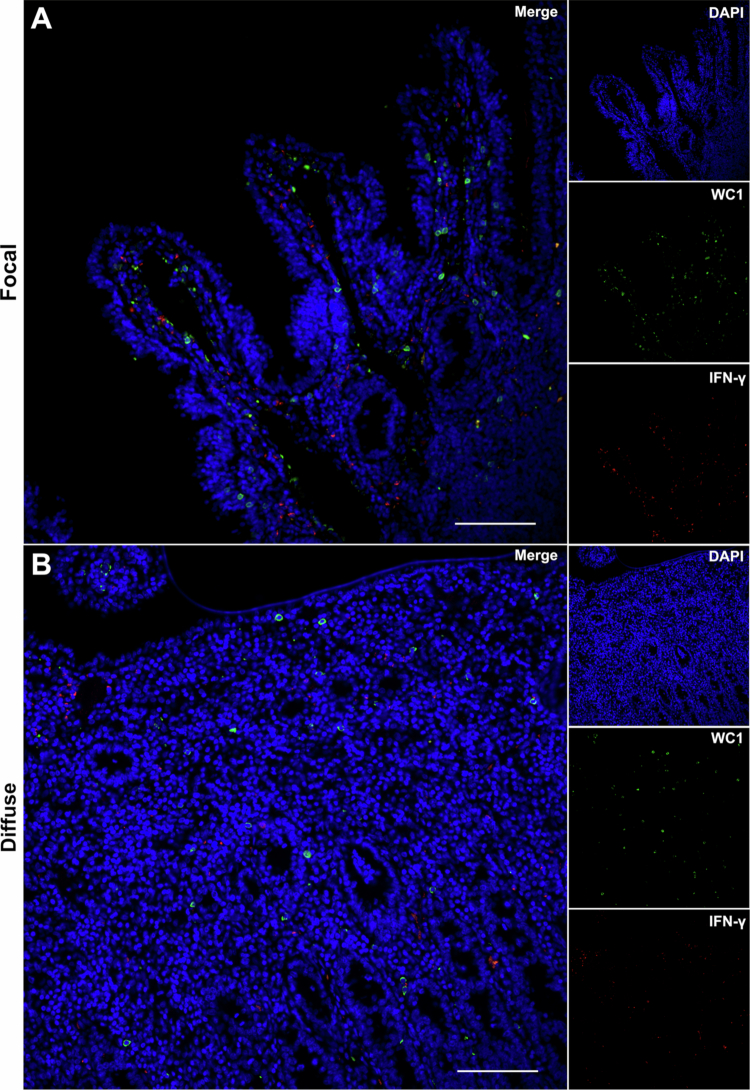
Double immunofluorescence labeling against WC1^+^Gamma delta T- lymphocytes (WC1) and interferon γ (IFN-γ). Distal jejunum Peyer's patch sections were labeled using DAPI (blue), an anti-WC1 monoclonal antibody with a secondary anti-mouse AF488 (green), and an AF647-labeled anti-IFN-γ antibody (red). Few WC1^+^ cells were colocalized with IFN-γ in animals with both (A) focal and (B) diffuse lesions. Micrographs were taken at 200×. Scale bar = 100 µm.

The double immunofluorescence analysis revealed that, in the studied animals, WC1 γδ T lymphocytes showed minimal co-localization with IFN-γ immunoreactivity (Figure 6). Whereas most cells positive to IFN-γ were WC1^−^ and morphologically compatible with lymphocytes and neutrophils.

### 3.4. IgA and IgA-producing plasma cells detection and distribution in the intestinal mucosa

**Figure 7.**
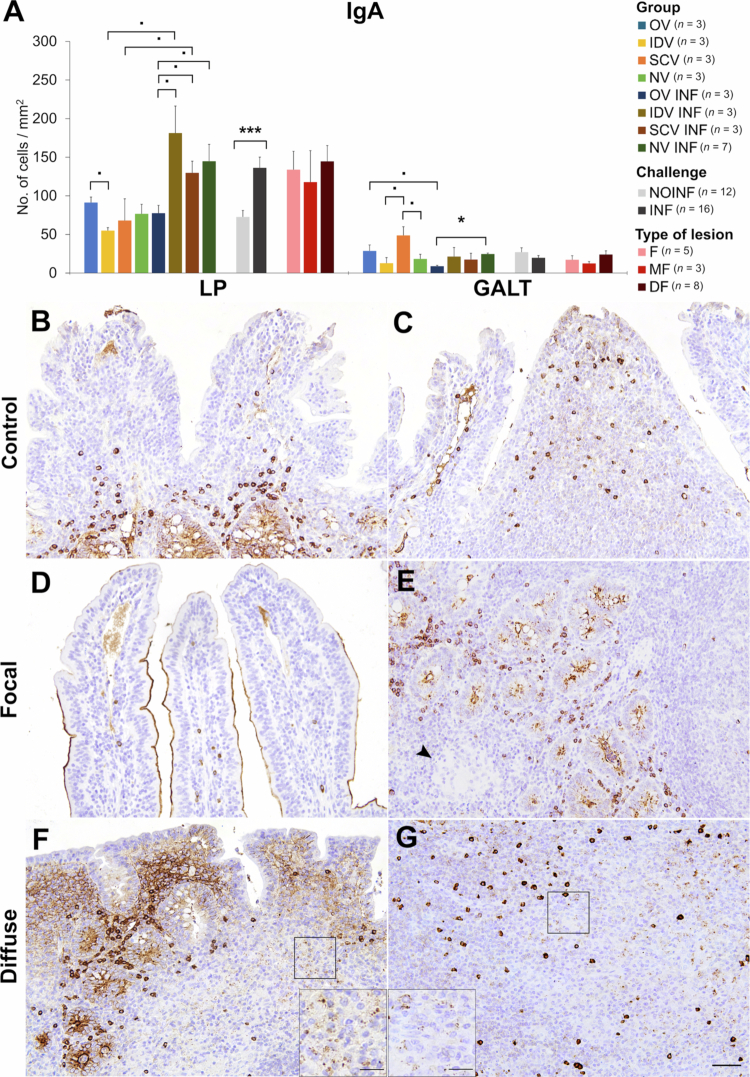
Numbers and distribution of the IgA^+^ in the distal jejunum Peyer's patches. (A) Mean cell counts of the immunolabeled cells present in the mucosa. All values are expressed as means and error bars represent the standard error. Brackets indicate statistically significant differences between groups. The dots indicate a *p* ≤ 0.1; *p < 0.05, ***p < 0.001. Photomicrographs of representative sections from the lamina propria (LP) (B, D, F) and gut-associated lymphoid tissue (GALT) (C, E, G). (B, C) Control animals and (D, E) Animals with focal lesions, showed similar IgA staining patterns, with higher abundance of IgA+ plasma cells in the (B) crypt region and (C) Peyer's patches domes. In some regions (D), extracellular IgA could be observed along the ciliated border of epithelial cells and within the lymph lacteals, and (E) no changes in the staining pattern were noted in association with focal granulomas (arrowhead). (F, G) Animals with diffuse lesions showed intense extracellular IgA staining in association with the granulomatous infiltrates of the LP in some regions, together with punctate immunostaining within macrophages (insets). Abbreviations for the different groups are indicated in Table 1. All images were acquired at the same magnification (200×). Scale bar = 50 µm, except for insets (scale bar = 20 µm).

The number of IgA-producing plasma cells was significantly increased (*p* < 0.001) in the LP of challenged animals (Figure 7A). And among experimental groups, its levels were lowest in both LP and GALT in the OV-INF group. In all the experimental groups, IgA^+^ plasma cells were abundant in the LP across all the animals, particularly in the crypt area (Figure 7B). In certain regions, IgA were also detected along the ciliated border of epithelial cells, and free IgA were observed within the lymph inside the lacteals (Figure 7D). Additionally, IgA^+^ plasma cells were prevalent in the dome of the PP (Figure 7C). However, these cells were sparse throughout the rest of the GALT. In the animals with focal lesions, the distribution pattern of IgA^+^ plasma cells remained like that observed in non-infected animals both in the LP and GALT, with no significant alterations associated with the presence of focal granulomas (Figure 7E). In animals with diffuse lesions, IgA^+^ plasma cells were irregularly distributed, particularly in areas where lesions were more severe, and in some of these areas, IgA were abundant in the extracellular spaces of the LP (Figure 7F). In the GALT, where diffuse lesions were present, there were higher numbers of IgA^+^ plasma cells (Figure 7G). In both LP and GALT, macrophages within the granulomatous infiltrates occasionally exhibited a punctate IgA staining (Figure 7F and G).

**Figure 8.**
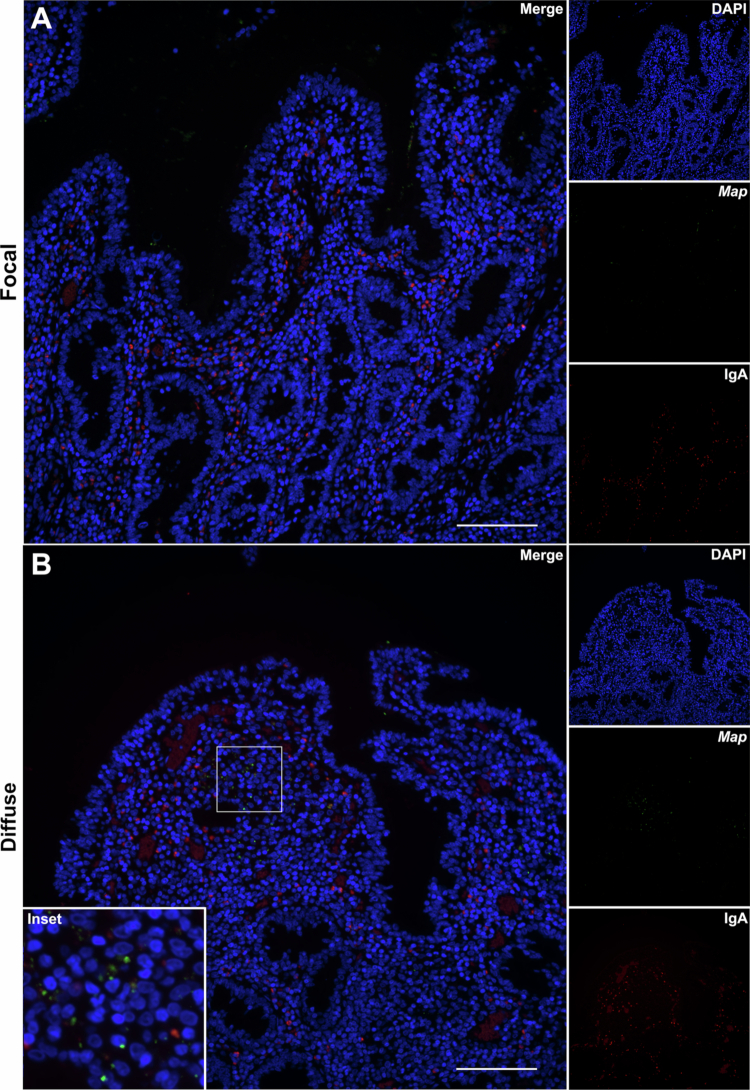
Double immunofluorescence labeling against *Mycobacterium avium* subspecies *paratuberculosis* (*Map*) and immunoglobulin A (IgA). Distal jejunum Peyer's patch sections were labeled using DAPI (blue), an anti-*Map* polyclonal serum with a secondary anti-mouse AF488 (green), and an AF647-labeled anti-IgA antibody (red). (A) In animals with focal lesions, *Map* could not be detected, and IgA^+^ plasma cells were abundant in the lamina propria, with free IgA being detected mainly in the lacteals of some of the villi. (B) In those with diffuse lesions, IgA^+^ plasma cells could also be observed throughout the lamina propria and, in the areas with marked lesions, where *Map* could be detected (inset), IgA were often present in the extracellular spaces. Micrographs were taken at 200×. Scale bar = 100 µm.

Double immunofluorescence staining revealed that, in diffuse lesions where *Map* was detected within the macrophages of the granulomatous infiltrate, abundant IgA was frequently observed in the extracellular matrix (Figure 8). Also, some macrophages loaded with both *Map* and IgA could be detected in the animals with diffuse lesions (Additional File 6).

### 3.5. Differential transcription of cytokines

**Figure 9.**
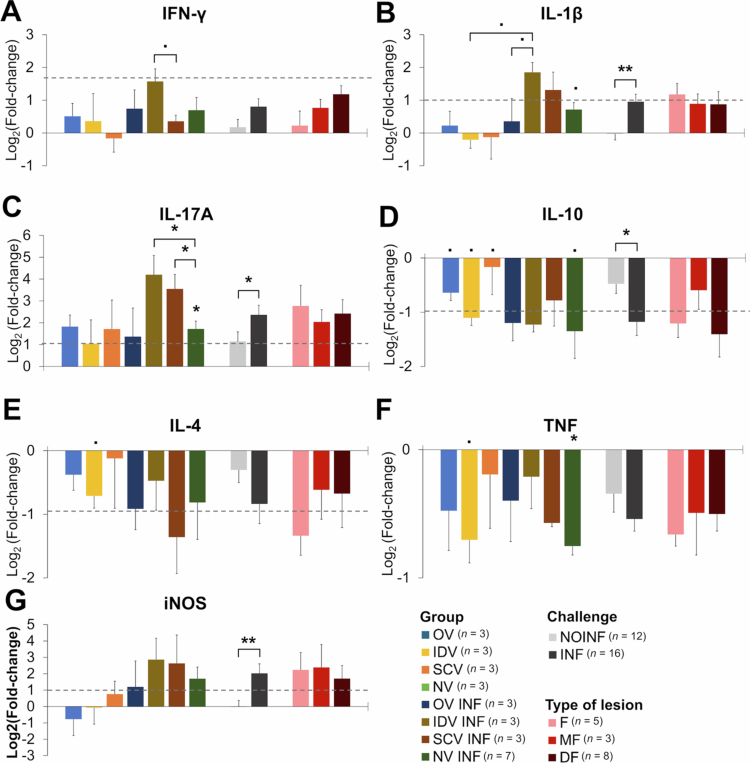
Differential transcript expression of cytokines and iNOS in DJPP. Bar plots represent the mean log_2_‑fold change (log_2_FC) in gene expression, as determined by qRT‑PCR, for the genes: (A) IFN-γ, (B) IL‑1β, (C) IL-17A, (D) IL-10, (E) IL-4, (F) TNF and (G) iNOS. The error bars represent the standard error. The dashed lines denote two-fold increases (FC = 2, log_2_FC = 1) or decreases (FC = 0.5, log_2_FC = −1) in gene expression with respect to the mean FC of the NV group. The symbols above the bars indicate significant differences with the NV group, and the brackets indicate statistically significant differences between groups. The dots indicate a *p* ≤ 0.1; *p < 0.05; **p < 0.01. Abbreviations for the different groups are indicated in Table 1.

IFN-γ, IL-1β and iNOS, cytokines associated with the Th1 immune response, were upregulated to variable degrees in most challenged groups (Figure 9A–F). Conversely, in these groups, the Th2 cytokines IL-4 and IL-10 were downregulated (Figure 9A–F). These transcriptional changes can be seen more clearly when grouping infected animals. The changes in TNF transcription were mild in all groups, and its expression was weakly correlated with that of IL-4 (correlation coefficient = 0.38) and IL-10 (correlation coefficient = 0.44) (Additional File 7), which is in line with that observed in the FAMD analysis (Figure 1). Regarding the Th17 response, IL-17A showed the highest relative transcription levels, which was at least doubled (log_2_FC > 1) in all groups. This denotes an influence of both vaccination and infection on the expression of this cytokine. IL-17A expression was particularly elevated in the IDV-INF and SCV-INF animals, showing changes exceeding 10-fold (log_2_FC = 3.2) in some cases.

### 3.6. IFN-γ and IL-4 production by DJPP-isolated mononuclear leukocytes stimulated with aPPD

**Figure 10.**
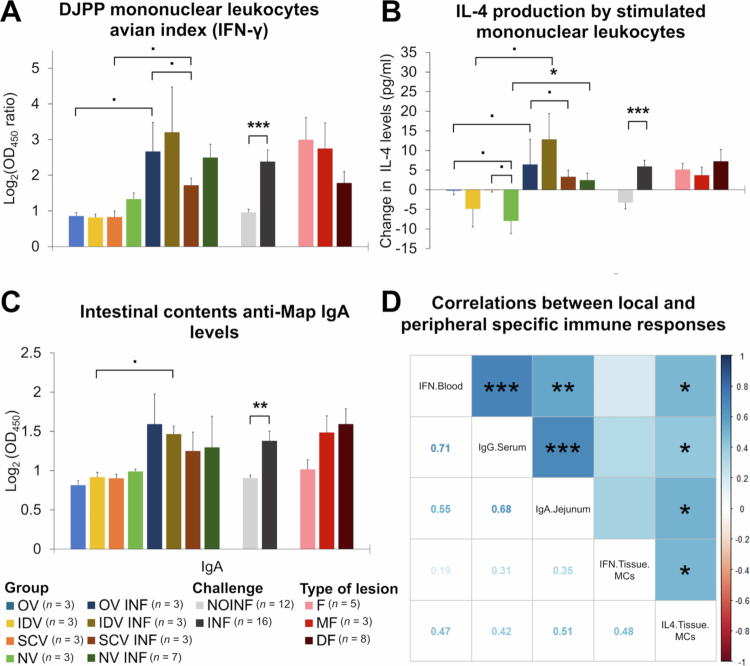
Local *Map*-specific cellular and humoral responses, as determined by ELISA, and their correlation with peripheral responses. Mean changes in (A) IFN-γ and (B) IL-4 production by *M. avium* purified protein derivative (aPPD)-stimulated mononuclear leukocytes isolated from a distal jejunum Peyer's patch (DJPP). IFN-γ results are expressed as the log_2_-transformed ratio between aPPD-stimulated and nil-stimulated (PBS) cultures. IL-4 results are expressed as the difference between aPPD-stimulated and nil-stimulated (PBS) cultures. (C) Mean anti-*Map* IgA levels in the jejunal content. (D) Matrix of correlations between the local and peripheral specific immune responses. Abbreviations for the groups are provided in Table 1, and for the variables, in Table 2. Values represent Spearman correlation coefficients (*ρ*), with colors indicating the direction and strength of the correlation (blue: positive; red: negative). Asterisks represent BH-adjusted correlation significances (*n* = 28). All values are expressed as means and error bars represent the standard error. Brackets indicate statistically significant differences between groups. The dots indicate a *p* ≤ 0.1; *p < 0.05; **p < 0.01; ***p < 0.001.

The mean production of IFN-γ and IL-4 by DJPP-isolated mononuclear leukocytes stimulated with aPPD was higher in all challenged groups, with respect to their non-challenged counterparts, and even though no significant differences were detected between the experimental groups, the SCV-INF showed the lowest production among the challenged groups (Figure 10A and B). In both cases, the differences were significant when grouping the animals by their infection status (*p* < 0.001). The production of both cytokines was moderately correlated (correlation coefficient: 0.47, *p* < 0.05). Regarding the peripheral IFN-γ response (IGRA), it was weakly and not significantly correlated with local IFN-γ production (correlation coefficient: 0.19) but was moderately correlated with local IL-4 production (correlation coefficient: 0.47, *p* < 0.05) (Figure 10D).

### 3.7. Quantification of *Map-*specific IgA in intestinal content

Results from the IgA ELISA are represented in Figure 10B. *Map-*specific IgA levels in the intestinal content were significantly higher in challenged animals (*p* < 0.01). In this regard, IgA levels in the jejunum were strongly correlated with *Map* loads in both feces (correlation coefficient = 0.761, *p* < 0.001) and DJPP (correlation coefficient = 0.693, *p* < 0.001). Although IgA levels were higher in all challenged groups compared to their non-challenged counterparts —with the OV-INF group showing the highest mean values—, these differences were not statistically significant.

Interestingly, in contrast with that observed with the local and peripheral IFN-γ responses, IgA levels showed a positive and highly significant correlation (correlation coefficient: 0.68, *p* < 0.001), with the specific-IgG levels in serum by the endpoint of the experiment and to a lesser extent with the peripheral cellular immune response, measured with the IGRA test (correlation coefficient: 0.55, *p* < 0.01) (Figure 10D).

## 4. Discussion

Current vaccines for controlling *paratuberculosis* are often developed and improved via stepwise experimentation, despite a lack of understanding of the mechanisms underlying their effectiveness (Purdie et al. 2022). Understanding these mechanisms would be essential for rational vaccine development. In this context, the samples used in the current study originated from a previous work (Criado et al. 2024) where the efficacy of experimental OV and IDV vaccines was compared with that of Gudair®. The OV reduced lesion severity, and the IDV reduced the bacterial load, although both were outperformed by Gudair®.

The present study investigated the mucosal (intestinal) immune responses established in the same animals, incorporating selected findings from previous studies to support and strengthen the conclusions drawn. The results suggest that ten months after infection, lesion severity is the main factor influencing the local response established against *Map*. However, the initial influence of vaccination, which is responsible for the subsequent development of lesions, could have faded at this timepoint, although increasing the sample number could help to prove this. Thus, given the high inter-individual variability in PTB, larger cohorts including both focal and diffuse lesion phenotypes per vaccine would strengthen conclusions. In any case, the findings might help to understand the long-term effects of vaccination and vaccination route on the local immune response against *Map*. This response has been scarcely studied in naïve animals, particularly goats, but even fewer studies have addressed the impact of vaccination on this mucosal response. Additionally, despite being approximately ten times greater in length than the ileum and containing 25–40 discrete PP, the mucosal response in the jejunum has been less studied than that in the ileum, as observed in Additional file 1. This is probably because IPP has been traditionally considered the portal of entry of *Map*, even though evidence suggests that both ileal and jejunal PPs may serve similar roles (Facciuolo et al. 2016, 2020). In fact, in the animals analyzed in this experiment (Criado et al. 2024), DJPP showed the highest mean granuloma counts and bacterial burden among all the studied tissues. This is in line with previous experimental infections in goats, which have shown that *Map* initially targets the JPP and associated lymphoid tissues, with progressive lesion development and increasing variability among animals by 12 months post-infection (Krüger et al. 2015; Köhler et al. 2015).

As summarized in Additional file 1, previous studies in cattle and sheep describe heterogeneous local cytokine expression patterns in PTB, with no fully consistent pattern across tissues or experimental settings, with no consistent pattern across tissues species, or lesion phenotypes (Alzuherri et al. 1996; Lee et al. 2001; Coussens et al. 2004; Khalifeh and Stabel 2004; Tanaka et al. 2005; Smeed et al. 2007; Kumar et al. 2010; Magombedze et al. 2015; Sonawane and Tripathi 2016; Roussey et al. 2016; Facciuolo et al. 2020). Increased expression of pro-inflammatory mediators such as IFN-γ, TNF, IL-1β, IL-6, and IL-17A is frequently observed, often accompanied by concomitant upregulation of regulatory cytokines, including IL-10 and TGF-β, which is consistent with a mixed inflammatory–regulatory environment characteristic of chronic infection. Importantly, this variability appears to be strongly influenced by lesion phenotype, as both paucibacillary and focal forms show distinct cytokine signatures compared with diffuse lesions, contributing substantially to inter-study differences. Additional discrepancies likely reflect variation in infection stage, host species, tissue compartment analyzed, and experimental versus natural infection models. In this context, our observations in the DJPP are consistent with studies reporting active inflammatory responses at sites associated with high bacterial burden, supporting the concept that tissue-specific pathology and lesion phenotype are key determinants of the local immune response in PTB. Therefore, the DJPP represented the most suitable tissue for immunological assessment in this model.

Regarding the vaccine effect, both the FAMD analysis and the evaluation of individual variables indicate that mucosal responses were largely comparable among unchallenged animals at this time point, even after a recall stimulus (ex vivo aPPD stimulation of tissue-isolated mononuclear cells). However, one exception was the relative expression of IL-17A which was increased in all vaccinated and unchallenged groups. Previous studies on mucosal vaccination against *M. tuberculosis*, have associated the presence of IL-17A-producing CD4^+^ T cells in the lungs with vaccine-induced protection (Gopal et al. 2013; Hammarlund et al. 2017). In this sense, memory Th17 cells have been previously described to last for up to two years in mice that were immunized parenterally against *M. tuberculosis* (Lindenstrøm et al. 2012). In PTB vaccinated animals, increased IL-17 responses after antigen stimulation have been reported across multiple PTB vaccine platforms (Faisal et al. 2013; Gupta et al. 2023). This increase was also observed after stimulation with a *Map* lysate of mononuclear cells isolated from the IPP of cattle vaccinated with an oral vaccine and challenged with *Map* (Eshraghisamani et al. 2024). In that study, a non-significant increase in the expression of IL-17 was also found in the JPP. However, overall, the response of tissue-isolated lymphocytes and RNAseq analysis revealed a lower responsiveness against *Map* of the JPP when compared to the IPP.

When analyzing the vaccinated and challenged animals, IL-17A levels were increased in the IDV-INF and SCV-INF groups with respect to their non-challenged counterparts, whereas no such increase was observed in the OV-INF group. A similar pattern was also observed when analyzing IL-1β expression. In this context, the reduced expression of IL-17A and IL-1β in the OV-INF group may have contributed to the relatively small number of lesions observed, despite their elevated local bacterial loads. These cytokines are essential for controlling mycobacterial infections but are also implicated in lesion development, as they can induce excessive inflammation, which leads to tissue damage (Lamont et al. 2012; Stewart et al. 2024). IL-1β is primarily produced by APCs and is essential for the development of Th17- and IL-17-secreting γδ T cells. In turn, the IL-17 produced by T cells can also activate macrophages and neutrophils (Lee et al. 2010; Milli 2023).

In parallel, this pattern may also be consistent with differences in antigen sampling and presentation associated with the oral route of vaccination, which could shape a mucosal immune environment that favors bacterial persistence without effective sterilizing immunity. Although the mechanisms hypothesized in our previous work (Criado et al. 2024), such as oral tolerance or T-cell exhaustion, could theoretically explain the reduced lesion severity observed in orally vaccinated animals, despite elevated bacterial burden, our data do not support a state of generalized immune hyporesponsiveness. In particular, the preservation of antigen-specific IFN-γ responses upon re-stimulation indicates that memory T-cell function remains intact (Mowat 2003; Wherry 2011). Collectively, these findings support the concept that, in this model, lesion severity is more tightly linked to host-driven immunopathology than to the bacterial load per se and that oral vaccination may partially uncouple inflammatory lesion development from the bacterial burden within intestinal tissues. The cytokine interplay which is taking place, and the cells involved in it, demands further research.

The observed differences in most variables of the local immune response were more strongly associated with animal infection status and lesion severity in the studied samples, rather than with the experimental group to which they belong, or the vaccine received. This aligns with the findings from the FAMD, which revealed that lesion severity and bacterial burden cause an increase the number of all the cell populations studied in the GALT and the numbers of Iba1 and M2 macrophages in the LP. It also showed a relationship between *Map* burden and both the local (IgA), and peripheral (IgG) humoral immune responses.

Corroborating these observations, when analyzing the numbers of total macrophages—and their M1 and M2 subpopulations—, they were elevated in the jejunal mucosa of the infected animals. As expected, the total macrophage numbers were higher in diffuse lesions, given that they are the primary component of the granulomatous infiltrate. Among the challenged groups, the OV-INF animals showed the lowest numbers of total macrophages in both the LP and GALT. Despite their markedly high bacterial burden, the number of granulomas was relatively low, higher than that in SCV-INF but lower than that in non-vaccinated or IDV-INF animals. This imbalance between the bacterial load and the granulomatous response suggests an inefficient local containment of the infection, possibly reflecting impaired activation and/or recruitment of macrophages within the intestinal mucosa. Increased CD163+ macrophages indicate a shift toward M2 polarization, which is consistent with previous reports in cattle (Fernández et al. 2017). However, animals with diffuse lesions also showed the highest numbers of M1 macrophages in the GALT, and they were also increased in the LP with respect to the non-challenged animals. This contrasts with previous findings in naturally infected cattle with diffuse lesions (Fernández et al. 2017). The relatively short post-infection period of 10 months in this study could mean that the immune response was still in transition, with the M1/M2 balance not yet fully skewed towards M2. However, an abundance of iNOS^+^ macrophages infiltrating diffuse lesions has been previously described in naturally infected goats (Agulló-Ros et al. 2022), so it could represent a species-specific difference. In the animals with focal lesions, large numbers of macrophages intensely labeled against iNOS could be seen in the periphery of the granulomas located in the GALT, but they were absent in most of the non-affected tissue. This localization suggests that despite their lower overall numbers, iNOS^+^ macrophages may be strategically positioned in areas with *Map* presence, where they could contribute to their elimination without causing excessive tissue damage. Also, all vaccinated and challenged animals (OV-INF, IDV-INF and SCV-INF) showed a higher number of iNOS^+^ macrophages in the LP, although this increase was not statistically significant. This finding has been previously described in cattle vaccinated with Silirum® (Zapico et al. 2025) and in macrophages isolated from goats vaccinated with Silirum® (Arteche-Villasol et al. 2021). The WC1^+^ γδ T cell number was also elevated in the GALT of animals with focal and diffuse lesions. These cells were in high numbers in the periphery of focal granulomas, as previously described in early BCG-induced granulomas (Kim et al. 1996), and in cattle naturally infected with *Map* displaying focal lesions (Criado et al. 2020), its possible role will be discussed further on.

Regarding secretory IgA, previous studies have shown that its quantification in feces or other samples, such as rectal secretions and saliva, is a poor predictor of intestinal IgA levels (Ferguson et al. 1995; Externest et al. 2000). In sheep exposed to, or infected with *Map,* the IgA release in feces was generally low and very irregular (Begg et al. 2015), although it has been demonstrated that IgA against *Map* are produced in large quantities in the jejunal PP (Facciuolo et al. 2016). Therefore, jejunal content samples, rather than fecal samples, seem ideal for studying the local humoral response and, in the present study, IgA levels were increased in the animals with the most severe lesions and were correlated with fecal and DJPP *Map* loads. On the other hand, in animals with focal lesions, specific IgA levels were low, close to the OD_450_ of non-challenged animals. This, together with the observation of accumulations of extracellular IgA in the periphery of diffuse lesions, could indicate that local IgA production is triggered by the local presence of *Map*.

The generation of *Map-*specific IgA-producing plasma cells could be initiated by the presence of free antigen in the lumen or extracellular matrix. In this sense, a strong correlation between intestinal IgA and serum IgG levels was observed. Since the onset of IgG production is a well-established marker of PTB progression (Vázquez et al., 2020), these findings suggest that local IgA responses may increase in parallel with antigen availability and lesion development. Further research should aim to establish the precise onset of secretory IgA production after PTB infection. Also, it would be of interest to study the putative induction of local IgA production by different vaccines at earlier timepoints. We have also observed that some *Map*-loaded macrophages contained IgA, suggesting that the mycobacteria may have been previously opsonized and phagocytosed. Overall, the production of this immunoglobulin could significantly influence the initial mucosal response at the infection site. For instance, a previous work demonstrated that the presence of antibodies from *Map-*infected cows significantly reduced *Map* invasion in an intestinal loop model (Jolly et al. 2022). Additionally, IgA levels in the intestinal content could constitute a reliable biomarker of disease progression or vaccine efficacy under experimental conditions. Liu et al. (2024) recently reported an increase in IgA^+^ cells and non-specific IgA secretion in mice orally vaccinated with different pathogen-mimicking nanoparticles; these vaccines reduced the bacterial load and granulomatous inflammation in the liver after *Map* challenge. In the present experiment, an increase in specific IgA was observed in the OV-INF animals, whereas the number of total IgA^+^ plasma cells was the lowest among the challenged groups. This finding could be explained by its production in upper portions of the jejunum or the induction of *Map*-specific IgA⁺ plasma cells in extraintestinal sites, such as the jejunal lymph nodes —where *Map* loads were high in this group—, and their subsequent migration to the intestinal LP to secrete *Map-*specific IgA. The scarcity of IgA⁺ plasma cells in the LP, despite high antigen-specific IgA in the lumen, may reflect impaired recruitment or survival within the mucosa due to the low expression of IL-1β and IL-17A, which have been implicated in mucosal IgA induction and IgA⁺ plasma cell development and maintenance (Hirota et al. 2013; Jung et al. 2015), and their low expression could plausibly impair plasma-cell retention or survival.

Further analysis of the FAMD results shows a correlation between the local expression of IL-10, IL-4, and TNF. These cytokines were downregulated in infected animals, however, the relative changes in expression were generally low. Several variables were negatively correlated with the expression of these cytokines. IL-1β was increased in infected animals, particularly in those with focal lesions. This cytokine is implicated in macrophage recruiting, activation and M1 polarization, as well as in the polarization of CD4^+^ T lymphocytes to a Th1 phenotype (Kaneko et al. 2019). In animals with focal lesions, this M1/Th1 polarization should imply a better infection control (Fernández et al. 2017). In these animals, M1 macrophages were increased in the LP even though no visible lesions were present in this area, which suggests an increased macrophage activation. However, this cytokine was also upregulated in animals with more severe lesions, where the presence of a large number of inflammatory cells could be the origin of an elevated basal production of this cytokine. In this line, intestinal IFN-γ expression increased with lesion severity, however, upon stimulation of intestinal mononuclear leukocyteswith aPPD, IFN-γ production was lower, and IL-4 production higher than that in animals with focal lesions, which may indicate Th2 polarization (Nakamura et al. 1997). This local IL-4 production correlated more strongly with peripheral IGRA responses than local IFN-γ production, suggesting that Th2 polarization may begin locally before becoming evident at the peripheral level.

In the FAMD variable contribution plot, the levels of IFN-γ expression and its production after aPPD stimulation were related to the number of WC1^+^ γδ T lymphocytes in the LP. This lymphocyte subset was elevated in the LP of animals with focal lesions, as previously described in cattle (Criado et al. 2020). Notably, one animal from the SCV-INF group showed particularly high values and exhibited no fecal shedding, as well as the lowest number of granulomas in the jejunum and DJPP among the challenged animals. This, and its location in the periphery of the focal granulomas in the GALT, would support its previously proposed role in early granuloma conformation and infection containment (Kim et al. 1996; Criado et al. 2020). Its antimycobacterial role and its implication in granuloma development could be mediated by IFN-γ production (Plattner et al. 2009; Criado et al. 2020). However, the double immunofluorescence assay revealed that, in the intestinal mucosa, few WC1^+^ γδ T cells seem to produce IFN-γ in both control and infected animals. Previous studies using peripheral blood lymphocytes have demonstrated that this cells produce IFN-γ in response to antigens from *M. tuberculos*is (Tsukaguchi et al. 1999), *M. bovis* (McGill et al. 2014) and *Map* (Albarrak et al. 2017). But only under 10% of WC1^+^ γδ T cells of *M. bovis* infected animals produce IFN-γ in response to *M. bovis* peptides (McGill et al. 2014). Low percentages have also been reported in PTB infected cattle, where approximately 6% of WC1.1^+^ T cells —a pro-inflammatory γδ T-cell subset strongly associated with IFN-γ production— produce this cytokine in subclinical animals, while a similar percentage of WC1.2^+^ cells —a less understood subset with heterogeneous or mixed cytokine profiles depending on the context— show IFN-γ production in animals with clinical disease (Albarrak et al. 2017; Damani-Yokota et al. 2018). Therefore, either these small number of IFN-γ producing γδ T cells would be producing large amounts of IFN-γ (Tsukaguchi et al. 1999), or their potential role could be mediated by the production of other cytokines.

As previously mentioned, WC1 γδ T lymphocytes were more abundant in the GALT of animals with diffuse lesions. This may be due to the presence of different subpopulations, one located in the epithelial layer and underlying mucosa of the LP, with innate-like properties (McCarthy and Eberl 2018), which could respond against *Map* presence participating in early granuloma formation. And other, mainly located in the GALT, which could be linked with the adaptive immune response (Rampoldi and Prinz 2022). In this sense, it is well known that γδ T cells exhibit considerable phenotypic and functional differences depending on the compartment in which they are present (Khairallah et al. 2018; Ullrich et al. 2021). For example, at the intestinal level, memory γδ T cells have been associated with an exacerbated inflammatory response mediated by the production of IL-17A (Khairallah et al. 2018). However, it must be noted that important differences between ruminants and other species exist regarding γδ T cells subsets and its distribution (Holderness et al. 2013). As with most variables, no significant effect of vaccination was observed on their numbers. However, in the LP of SCV-INF animals, there was an increase in WC1 γδ T lymphocytes, which could be one of the factors influencing the higher protective features of SCV, since γδ T cells have been suggested to play a role in protection against mycobacterial infections (Albarrak et al. 2017). Similarly, in cattle orally vaccinated against PTB, no significant effect on the numbers of this T-cell subset in the jejunal and IPP, in neither control or infected animals was found (Eshraghisamani et al. 2023).

The main conclusion of this work is that the extent of *Map* infection and the severity of lesions in a goat model are the primary drivers of the local immune response established against *Map,* exerting a stronger influence than vaccination at ten months post-challenge. In this context, when comparing the intestinal immune responses of naïve and vaccinated animals or animals vaccinated with different products, categorizing the animals based on the locally established lesions constitutes a useful approach. However, the limited sample size and variability constrain inference, and future studies should include larger cohorts and earlier or longitudinal sampling to better resolve vaccine effects, as well as study this response at an earlier time point, as has been done in recent studies in naïve animals (Köhler et al. 2015; Lee et al. 2023) and in the IPP (Facciuolo et al. 2020; Eshraghisamani et al. 2024). Examining vaccinated animals shortly after challenge—before infection is fully established—could yield valuable insights. And particularly, tracking the subsequent pathological evolution, using longitudinal techniques such as sequential biopsies or the gut-loop model (Facciuolo et al. 2020) would provide a deeper understanding of the mechanisms underlying vaccine-induced protection.

## Supplementary Material

Supplementary Material Supplementary_material.docx

## Data Availability

The data that support the findings of this study are available from the corresponding author upon reasonable request.
