# Supplementary material for: Jejunal mucosal immune response in goats ten months after Mycobacterium avium subsp. paratuberculosis challenge is primarily determined by lesion severity rather than vaccination route
Source: Vet Q. 2026 Jul 23;46(1):2707278. doi: 10.1080/01652176.2026.2707278 (PMC13403457; doi:10.1080/01652176.2026.2707278)
Supplement: Supplementary Material — Supplementary_material.docx [file TVEQ_A_2707278_SM7044.docx]

**Supplementary material**

**Additional file 1. Changes in the local cytokine expression induced by PTB infection reported in previous studies in ruminants.** Results are from animals with diffuse multibacillary lesions or clinical PTB, except where indicated by asterisks, which denotes animals with paucibacillary (*) or focal (**) lesions. Overall, an upregulation in the expression of most cytokines was reported in studied tissues: jejunum, jejunal Peyer’s patches (JPP), ileum, ileal Peyer’s patches (IPP) and mesenteric lymph nodes (MLN).

| **Species**  **(Reference)** | **Tissue** | | |
| --- | --- | --- | --- |
|  | **Jejunum/JPP** | **Ileum/IPP** | **MLN** |
| Cattle (33) |  | ↑ IFN-γ, TGF-β, IL-1β IL-5, IL-8  ↓ IL-16 | ↑ IL-1α, IL-8, IL-2, IL-10  ↓ TGF-β, IL-16 |
| Cattle (22) | ↑ IL-8, IL-4, IL-2, IFN-γ, IL-1α | ↑ IL-12, IL-17A, TGF-β, TNF, IFN-y, IL-1A |  |
| Cattle (34,35) |  | ↑ TGF-β, IL-10, IFN-γ * | ↑ TGF-β, IL-10, IFN-y* |
| Cattle (38) |  | ↑ IL-1α, IL-1 β, IFN-γ, IL-6 |  |
| Cattle (37) |  |  | ↑ IL-2, IL-4, IL-10  ↓ IL-18 |
| Cattle (36) |  | ↑ IFN-γ**, IL-1α**, IL-10**, TNF**  ↓ TGF-β | ↑ IFN-γ, IL-1α, IL-10, TNF |
| Sheep(40) |  |  | ↑ IFN-y, TNF, IL-4 |
| Sheep (42) |  | ↑ TGF-β, IL-10*, TGF-β*, IFN-γ*  ↓ IL-1α | ↑ IL-10, TGF-β, IL-10*  ↓ IFN-y |
| Sheep (39) |  | ↑ TNF, IL-1β, IL-6, TNF* |  |
| Sheep (41) |  | ↑ Traf-1, IL-8, TNF, TGF-β, Traf-1*, IL-8*, IL-12*, IFN-γ*, IL-1β TNF*, TGFβ* | ↓ IL-10 |

**Additional file 2.** **Primary antibodies, unmasking technique and dilution used for immunofluorescence.** Heat-induced epitope retrieval (HIER) was performed at 95 °C for 20 minutes using PT-Link and Dako Target Retrieval Solutions (Agilent Technologies, Santa Clara, CA, USA). *Conjugated with AF647, using Alexa Fluor® 647 Conjugation Kit (Fast) - Lightning-Link^®^ (Abcam, Cambridge, UK).

| Target | Clone/  Reference | Epitope demasking | Dilution | Supplier |
| --- | --- | --- | --- | --- |
| *Map* | Rabbit anti-*Map* sera | HIER,  pH 6.0 | 1:1,000 | In-house (25) |
| Cow IgA H&L | ab112630* |  | 1:250 | Abcam (Cambridge, UK) |
| Bovine WC1  (workshop cluster 1) | CC15/ MCA838GA | HIER,  pH 9.0 | 1:150 | Bio-Rad laboratories Inc. (Hercules, CA, USA) |
| Bovine IFN-γ  (interferon γ) | CC330/ MCA2112* |  | 1:25 | Bio-Rad laboratories Inc. (Hercules, CA, USA) |

**Additional file 3.** **Primary antibodies, unmasking technique and dilution used for immunohistochemistry.** Heat-induced epitope retrieval (HIER) was performed at 95 °C for 20 minutes using PT-Link and Dako Target Retrieval Solutions (Agilent Technologies, Santa Clara, CA, USA).

| Target antigen | Clone/  Reference | Epitope demasking | Dilution | Supplier |
| --- | --- | --- | --- | --- |
| Rat Iba1  (ionized calcium-binding molecule 1) | 019-19741 | HIER,  pH 6.0 | 1:2,000 | Wako (Japan) |
| Murine iNOS  (inducible nitric oxide synthase) | ABN26 | HIER,  pH 6.0 | 1:2,000 | Merck Millipore (Cork, Ireland) |
| Human CD163 | EDHu-1/ MCA1853 | HIER,  pH 6.0 | 1:300 | Bio-Rad laboratories Inc. (Hercules, CA, USA) |
| Bovine WC1  (workshop cluster 1) | CC15/ MCA838GA | None | 1:300 | Bio-Rad laboratories Inc. (Hercules, CA, USA) |
| Cow IgA H&L | ab112630 | HIER,  pH 6.0 | 1:1,000 | Abcam (Cambridge, UK) |

**Additional file 4. Sequences of primers used for qRT‑PCR and standard curve data**

| **Target^a^** | **Primer** | **Primer sequences (5’-3’)** | **Product size (bp)** | ***R*^2 b^** | **Slope^c^** | **Efficiency (%)** | **Reference** |
| --- | --- | --- | --- | --- | --- | --- | --- |
| TNF (NM_001024860.1)^d^ | QTNFα-Fw | CCAGAGGGAAGAGCAGTCC | 126 | 0.994 | -3.6 | 89.6 | (56) |
|  | QTNFα-Rv | GGAGCGCTGATGTTGGCTAC |  |  |  |  |  |
| IFN-γ  (X52640.1) | QIFN-Fw | GATTCAAATTCCGGTGGATG | 110 | 1 | -3.478 | 93.9 | (56) |
|  | QIFN-Rv | TTCTCTTCCGCTTTCTGAGG |  |  |  |  |  |
| IL-1β  (NM_001009465.2) | QIL1β-Fw | ACCCCAAAGTCTACCCCAAG | 99 | 0.995 | -3.776 | 83.9 | (58) |
|  | QIL1β-Rv | TGAGTCTGTCCTGTACCCTA |  |  |  |  |  |
| IL-4  (XM_004008636.1) | QIL4-Fw | CTGCCCCAAAGAACGCAACT | 154 | 0.993 | -3.739 | 85.1 | (56) |
|  | QIL4-Rv | TCATTCACAGAACAGGTCTTGCTT |  |  |  |  |  |
| IL-10  (NM_001009327.1) | QIL10-Fw | TGCTGGATGACTTTAAGGGTTACC | 60 | 1 | -3.479 | 93.8 | (56) |
|  | QIL10-Rv | AAAACTGGATCATTTCCGACAAG |  |  |  |  |  |
| IL-17A  (XM_004018887.4) | QIL17A-Fw | AGTCTGGTGGCTCTTGTGAA | 113 | 1 | -3.624 | 88.8 | (58) |
|  | QIL17A-Rv | TTAACGATGTTCAGGTTGAC |  |  |  |  |  |
| iNOS  (XM_013971952.2) | QiNOS-Fw | CTTTTGGCAACGGAGACTC | 125 | 1 | -3.358 | 98.5 | (58) |
|  | QiNOS-Rv | CTGAGGGTACATGCTGGA |  |  |  |  |  |
| β-actin  (NM_001009784.1) | QBACTIN-Fw | ACACCGCAACCAGTTCGCCAT | 216 | 0.988 | -3.241 | 103.5 | (56) |
|  | QBACTIN-Rv | GTCAGGATGCCTCTCTTGCT |  |  |  |  |  |
| GAPDH  (JN811680.1) | QGAPDH-Fw | CTGGCCAAGGTCATCCAT | 86 | 0.997 | -3.493 | 93.3 | (57) |
|  | QGAPDH-Rv | ACAGTCTTCTGGGTGGCAGT |  |  |  |  |  |
| ^a^ NCBI accession numbers are for *Ovis aries* or *Capra hircus* cDNA sequences used in primer design. When using ovine sequences, they were checked for similarities with caprine sequences using basic local alignment search tool BLAST.  ^b^ Mean minimum coefficient of regression (R2) of standard curves.  ^c^ Mean of standard curve slopes.  ^d^ GenBank accession number. | | | | | | | |


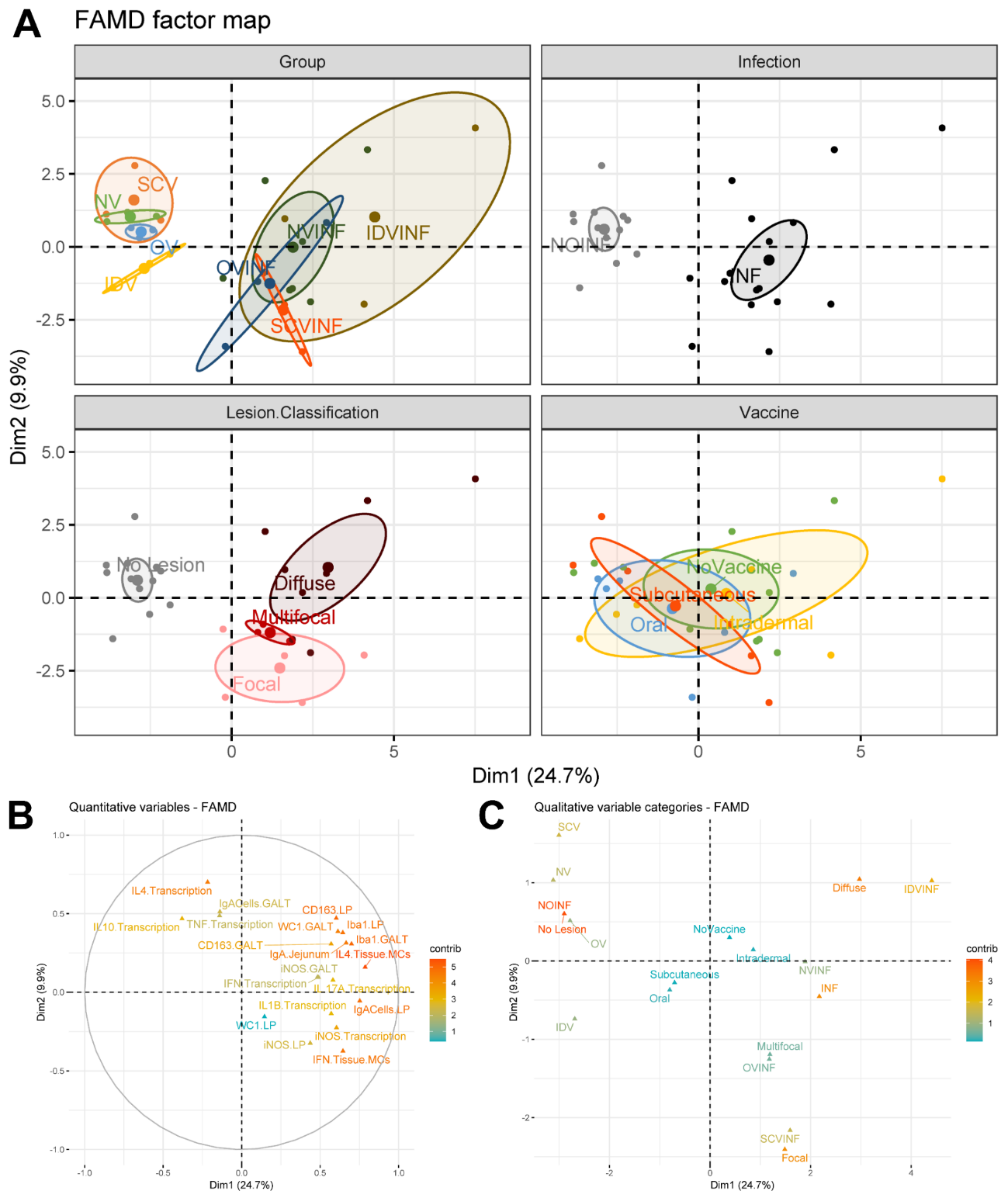


**Additional file 5. Factorial Analysis of Mixed Data (FAMD) of the local immune response.** **(A)** Elliptical factor map, individuals are indicated with a dot, colored based on their experimental group, infection status, lesion classification or vaccine received. The ellipses are drawn around the centroid (mean position) of each group and capture the spread and variability of the data points within that group. Its size reflects the concentration of the data; larger ellipses indicate greater variability among individuals in that group. **(B)** Quantitative variables contribution plot. **(C)** Qualitative variables contribution plot. Abbreviations for the groups are provided in *Table 1*, and for the variables in *Table 2*.


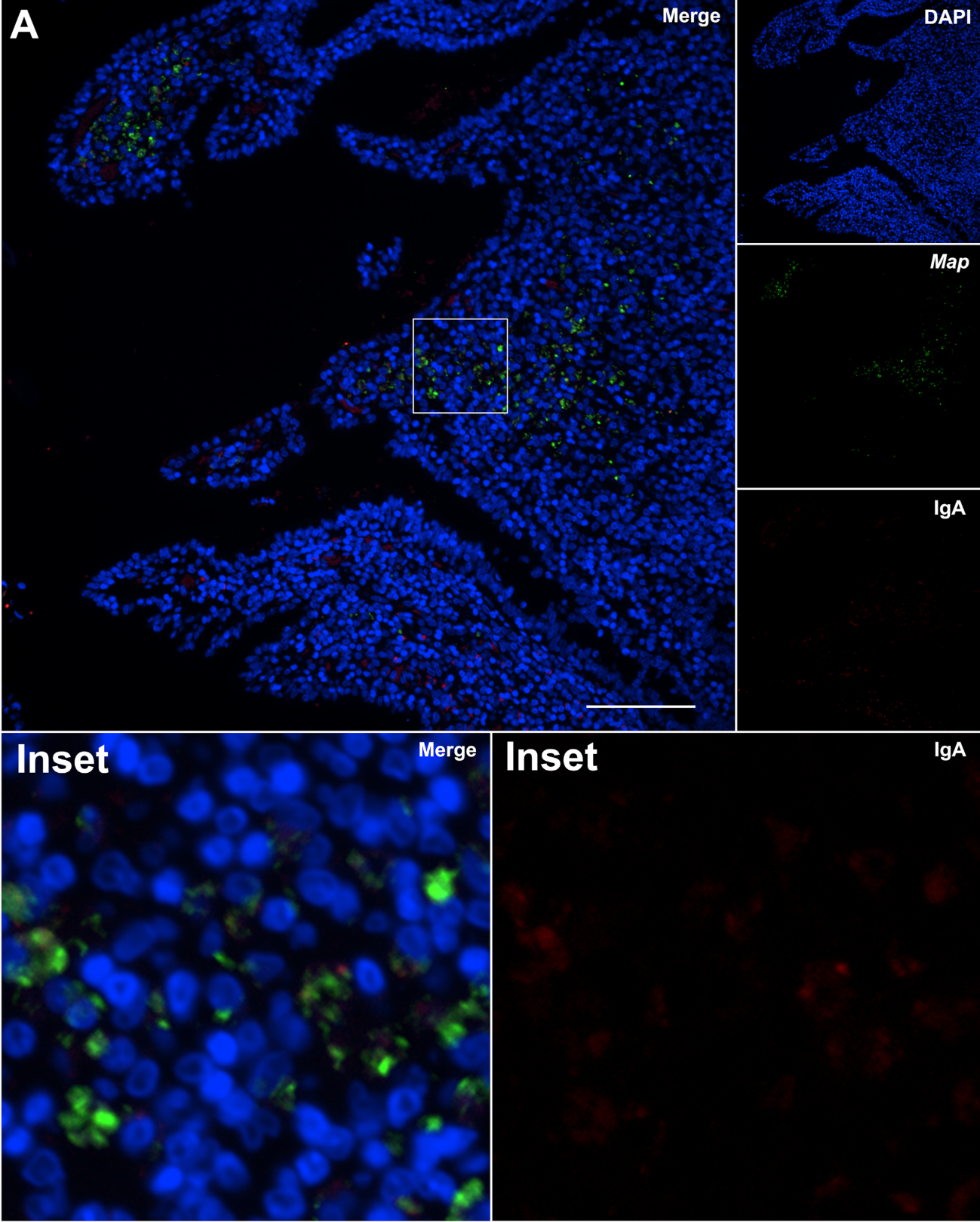


**Additional file 6. Double immunofluorescence labelling against *Mycobacterium avium* subspecies *paratuberculosis* (*Map*) and immunoglobulin A (IgA) in an animal with diffuse lesions.** The DJPP section was labelled using DAPI (blue), an anti-*Map* polyclonal sera with a secondary anti-mouse AF488 (green), and an AF647-labeled anti-IgA antibody (red). **(A)** Map-loaded macrophages are present within the diffuse granulomatous infiltrate. In the **(Inset)**, some macrophages are labeled for both Map and IgA. Micrographs were taken at 200 ×. Scale bar = 100 µm.


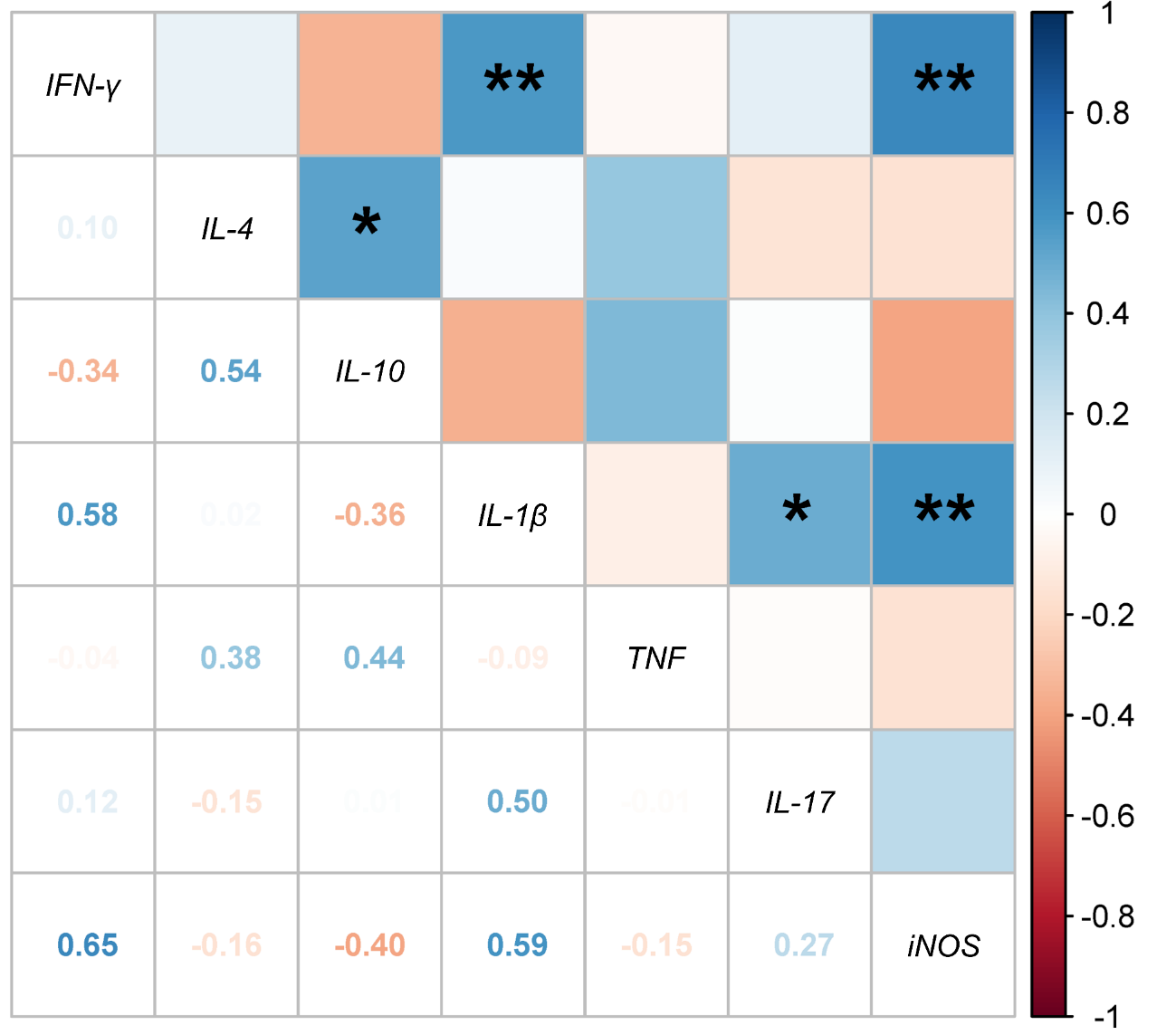


**Additional File 7. Differential transcript expression of cytokines and iNOS in DJPP.** Values represent Spearman correlation coefficients (ρ), with colors indicating the direction and strength of the correlation (blue: positive; red: negative; white: no correlation). Asterisks represent BH-adjusted correlation significances (*n* = 28). All values are expressed as means and error bars represent the standard error. Brackets indicate statistically significant differences between groups. * *p* < 0.05, ** *p* < 0.01.
